# Organic Dye-Modified Two-Dimensional Metal–Organic Framework/Carbon Nanotube Composite Films for Photothermoelectric Applications

**DOI:** 10.1021/acsnano.6c00103

**Published:** 2026-04-08

**Authors:** Cheng-Yuan Lin, Kuan-Chu Wu, Chih-Wei Hsu, Shao-Huan Hong, Chi-Lun Chuang, Te-Jen Hsu, Jhih-Min Lin, Chung-Wei Kung, Cheng-Liang Liu

**Affiliations:** † Department of Materials Science and Engineering, 33561 National Taiwan University, Taipei 10617, Taiwan; ‡ Department of Chemical Engineering, 34912 National Cheng Kung University, Tainan 701401, Taiwan; § 57815 National Synchrotron Radiation Research Center, Hsinchu 30076, Taiwan; ∥ Institute of Polymer Science and Engineering, National Taiwan University, Taipei 10617, Taiwan; ⊥ Advanced Research Center for Green Materials Science and Technology, National Taiwan University, Taipei 10617, Taiwan

**Keywords:** metal−organic framework, photothermoelectric, dye, carbon nanotube, thermoelectric, wearable, composite

## Abstract

Photothermoelectric (PTE) systems, which convert light into electricity through sequential photothermal (PT) and thermoelectric (TE) processes, offer a promising strategy for self-powered wearable electronics. In this work, we develop a homogeneous PTE composite system by integrating carbon nanotubes (CNTs) with a dye-modified two-dimensional metal–organic framework (2D MOF), referred to as ZrBTBD, obtained via the postsynthetic modification of a 2D MOF, ZrBTB, with N719 dye. The introduction of N719 enhances visible-light absorption and facilitates doping level modulation with CNTs. Together with n-type doping using N-DMBI, the resulting p-type C/ZrBTBD10 and n-type C/ZrBTBD-N5 composites achieve high power factors of 465.7 and 363.1 μW m^–1^ K^–2^, respectively. Under 100 mW cm^–2^ illumination, the PT temperature increases from 47.3 °C to 51.2 °C, and the *zT* is significantly enhanced compared to CNTs. A flexible PTE generator assembled from these composites delivers an open-circuit voltage of 12.3 mV and a maximum power output of 365.4 nW. A wearable prototype demonstrates its potential for flexible, self-powered electronics. This work represents the demonstration of dye-immobilized MOF/CNT composite materials in PTE systems, offering a molecular-level strategy for integrating light harvesting, interfacial charge modulation, and thermoelectric conversion.

## Graphical abstract

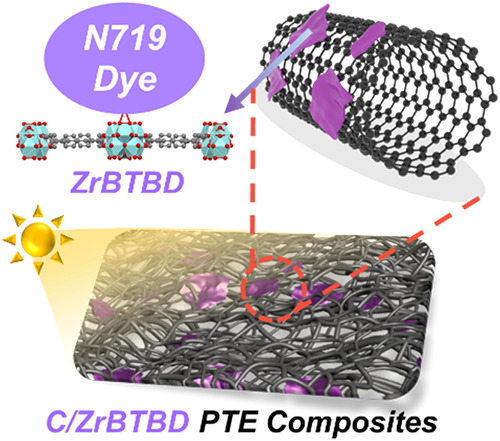

## 1 Introduction

Thermoelectric (TE) materials enable the direct conversion of thermal gradients into electrical energy and hold great promise for waste heat recovery and self-powered electronics. The thermoelectric figure of merit, *zT*, is a dimensionless parameter used to evaluate the heat-to-electricity conversion efficiency of a material, defined as

1 $$\mathrm{zT}=S^{2}\sigma T\kappa^{-1}$$

where *S* is the Seebeck coefficient, σ is the electrical conductivity, κ is the thermal conductivity, and *T* is the absolute temperature. An ideal TE material should simultaneously possess high *S* and σ values, while maintaining a low κ. However, these parameters are inherently interdependent, which poses a major challenge in optimizing the *zT*.^1^ To address this limitation, the photothermoelectric (PTE) effect has emerged as a powerful strategy, combining photothermal (PT) and TE conversion processes to provide enhanced energy harvesting efficiency.^2,3^ In PTE systems, incident light, (most commonly sunlight), is absorbed and rapidly converted into localized heat via nonradiative relaxation pathways, thereby generating temperature gradients that drive the TE response. A particularly promising approach to enhance PTE performance involves the development of *homogeneous* architectures, wherein a single material system simultaneously exhibits both PT and TE functionalities.^4^ Compared to heterogeneous systems, homogeneous PTE systems minimize interfacial thermal resistance, simplify device fabrication, and offer superior mechanical integration for highly desirable flexible and miniaturized energy systems.^5–7^ Among the available materials, carbon nanotubes (CNTs) are especially attractive due to their π-conjugated structure and one-dimensional morphology, which afford efficient light absorption and PT conversion. Furthermore, CNTs possess high electrical conductivity and allow for tunable carrier polarity (p- or n-type) through chemical doping or band structure modulation.^8–10^ For example, Wang et al.^11^ used automated laser processing to fabricate a CNT-based PTE device with integrated p- and n-type modules, thereby achieving a high power density of 0.32 μW cm^–2^ under 200 mW cm^–2^ illumination. However, pristine CNTs suffer from high thermal conductivity, which restricts the formation of a temperature gradient, along with relatively limited tunability of their optical absorption and electronic properties.^12^ To address these challenges, hybrid composite strategies have been increasingly explored that combine CNTs with functional materials to simultaneously enhance the PT conversion and TE performance.^13–16^

Metal–organic frameworks (MOFs) have attracted considerable interest as functional components in energy materials due to their molecular-level tunability, high porosity, and tailorable chemical functionalities in their pores.^17–24^ In particular, electrically conductive MOFs with intrinsically low thermal conductivities are appealing candidates for TE materials,^25–27^ while even insulating MOFs have been employed as functional additives in composite TE systems over the past decade.^28,29^ For example, our group recently reported the use of two-dimensional (2D) molecular sheets consisting of the zirconium-based MOF, ZrBTB (where BTB = 1,3,5-benzenetribenzoate),^30,31^ as a dispersant for CNTs to obtain composites with superior TE performances compared to either constituent alone.^32^ The Zr_6_ nodes in ZrBTB contain terminal −OH/OH_2_ ligands that are capable of postsynthetic modification, thereby providing a platform for further functionalization.^33–35^ Given the strong UV absorption of both the BTB linker and the CNTs, we reasoned that by coordinating carboxylate-based dye molecules to the nodes of ZrBTB, the resulting composite consisting of CNTs and dye-immobilized MOF should extend composite absorption into the visible range, thereby enhancing PTE performance. To this end, the ruthenium-based dye N719 was selected due to its broad and intense visible-light absorption and multiple carboxylic acid groups, which enable stable coordination with Zr nodes within the MOF framework. In addition, N719 has been reported to exhibit high photostability,^36^ which makes it an attractive dye for enhancing the PT conversion efficiency when integrated into CNT/MOF composites in this work. While previous studies have explored MOF/CNT composite systems, to the best of our knowledge, the use of dye-immobilized MOFs for PTE devices has not yet been reported.

Hence, the present study reports the development of a homogeneous PTE composite material by combining CNTs with dye-immobilized 2D Zr-based MOFs. Specifically, the ZrBTB framework is postsynthetically modified with the N719 dye to yield ZrBTBD, which not only broadens the absorption but also modulates the electronic interactions and doping level of CNTs. In addition, to achieve full p-n PTE integration, N-DMBI is introduced as an n-type dopant. The resulting p-type C/ZrBTBD10 and n-type C/ZrBTBD-N5 composites exhibit significantly improved TE properties, with power factors of 465.7 and 363.1 μW m^–1^ K^–2^, respectively, which represent the highest values reported thus far for CNT/MOF-based TE materials. Moreover, the incorporation of ZrBTBD not only leads to a significant reduction in thermal conductivity, thereby enhancing the *zT* relative to pristine CNTs, but also improves the PT conversion, as evidenced by an increase in surface temperature from 47.3 °C to 51.2 °C under 100 mW cm^–2^ illumination. Finally, a flexible 10-leg PTE generator (PTEG) assembled from these optimized composites demonstrates an open-circuit voltage of 12.3 mV and a maximum power output of 365.4 nW under simulated solar illumination. Remarkably, the output voltage per leg ranks among the highest reported for organic–inorganic hybrid homogeneous PTE systems. These results suggest the feasibility and efficacy of using MOF-based composites for high-performance, flexible, and wearable PTE energy-harvesting devices.

## 2 Results and Discussion

### 2.1 Synthesis and Characterization of MOF

First, the ZrBTB was synthesized by following the previously reported procedure.^37^ After that, solvent-assisted ligand incorporation (SALI) was performed in order to immobilize N719 molecules on the molecular ZrBTB sheets,^33,38^ as illustrated in Figure 1. Further experimental details are provided in the Supporting Information. The powder X-ray diffraction (PXRD) patterns of the resulting ZrBTB and ZrBTBD samples are presented in Figure 2a, where sharp diffraction peaks are observed at 5.1, 8.8, and 10.2°. These are consistent with the simulated pattern of ZrBTB, thereby indicating that the crystallinity of ZrBTB is preserved upon postsynthetic modification with N719. In addition, the nitrogen adsorption–desorption isotherms obtained at 77 K show that both materials possess similar Brunauer–Emmett–Teller (BET) surface areas of around 290 m^2^ g^–1^ (Figure 2b). It should be noticed that since the distance between adjacent stacked molecular sheets of ZrBTB is only around 0.7 nm,^31^ the immobilization of dye molecules on the nodes when these sheets were fully dispersed could expand the distance between sheets and thus increase the microporous space after these sheets stacked back, resulting in the slightly increase in the BET surface area. Similar observations after the postsynthetic modifications of ZrBTB have been reported in previous studies.^34,39^ In addition, hysteresis loops can be found in the high relative pressure range, which can be attributed to the spaces between aggregated MOF particles in the mesoporous scale.^34^ The pore size distributions of both materials were calculated from their isotherms by using density functional theory (DFT). As shown in Figure S1, the ZrBTB shows two primary pore sizes centered at 1.2 and 0.8 nm, which respectively relate to the size of the apertures on the planar 2D sheets and the interlayer spacing between stacked MOF sheets.^34^ By contrast, the ZrBTBD exhibits pore sizes of 1.0 and 0.8 nm, thereby indicating that the micropores on the 2D MOF sheets are partially occupied by the dye molecules after the SALI process.

**Figure 1:**
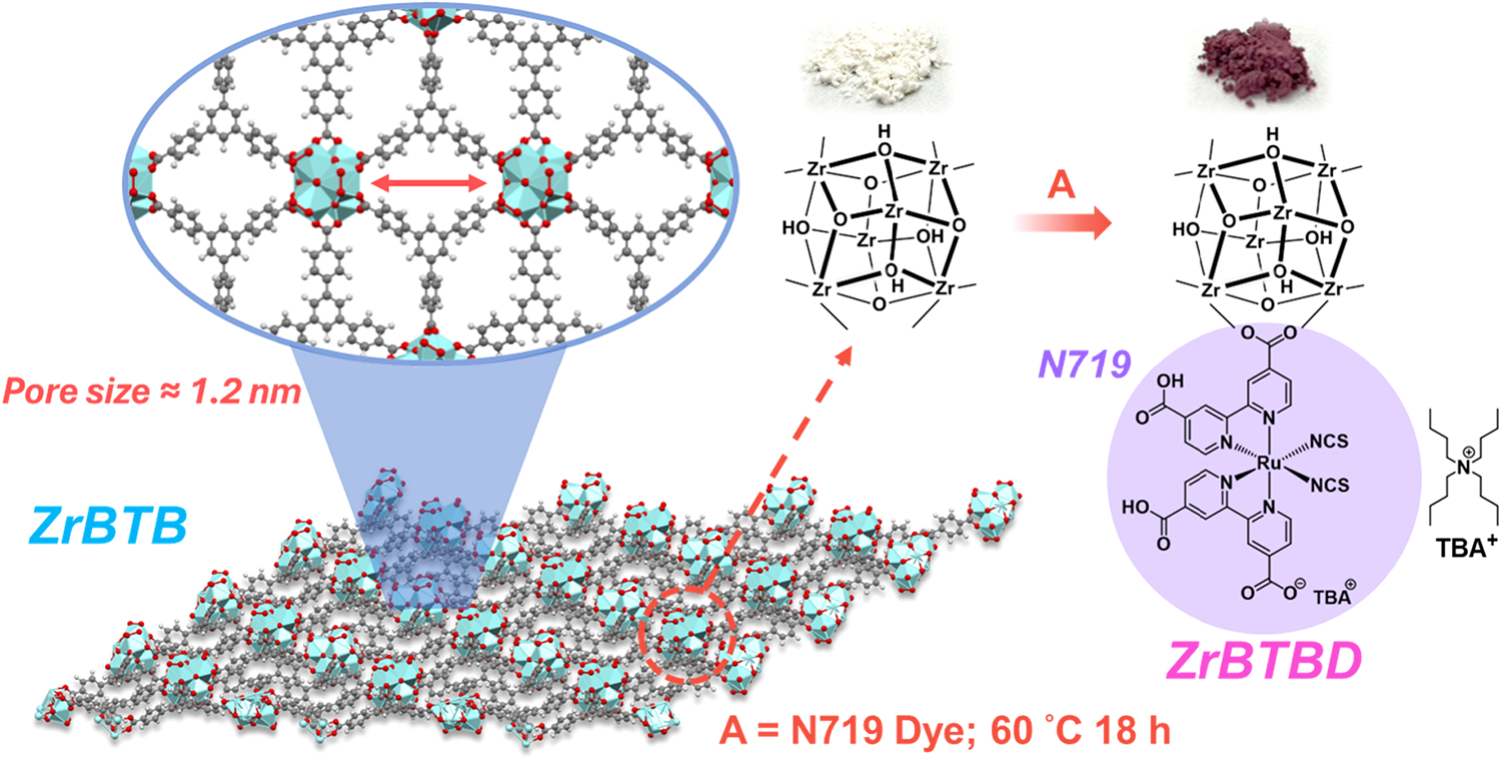
Schematic representation of N719 dye immobilization on ZrBTB to form ZrBTBD.

**Figure 2:**
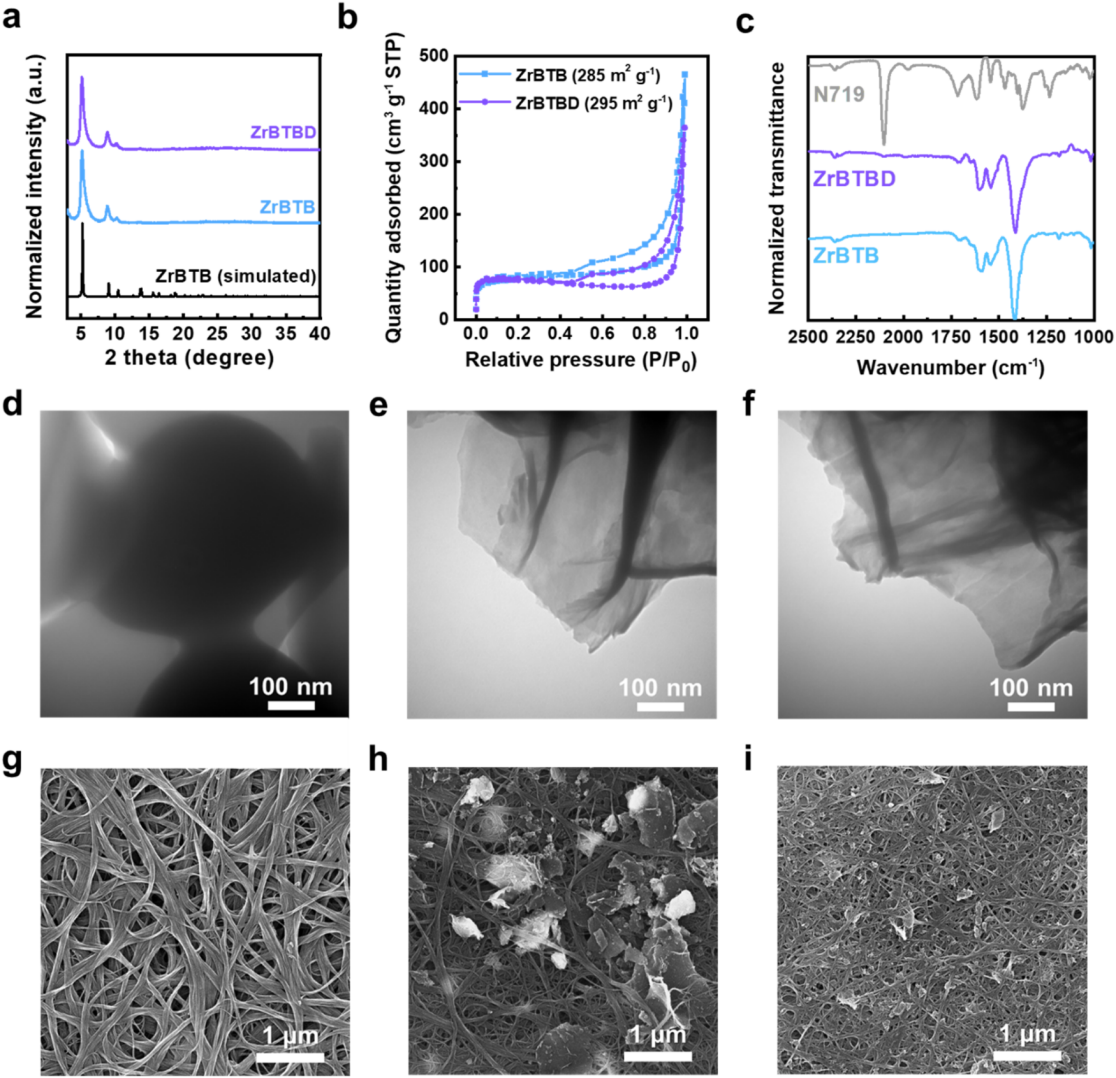
(a) PXRD pattern, (b) nitrogen adsorption–desorption, and (c) FTIR data of ZrBTB, ZrBTBD. The simulated pattern of ZrBTB and the N719 dye are shown in parts (a, c), respectively. High-magnification TEM of (d) N719, (e) ZrBTB, and (f) ZrBTBD. High-magnification SEM images of (g) CNT, (h) C/ZrBTB10, and (i) C/ZrBTBD10.

The Fourier transform infrared (FTIR) spectra of the ZrBTB and ZrBTBD samples are presented in Figure 2c, along with that of the pristine N719 dye. Here, the ZrBTB and ZrBTBD each exhibit peaks centered at 1412, 1540, and 1590 cm^–1^, which are associated with the −(O–C–O)– groups and aromatic C=C bonds of the ZrBTB,^34,40,41^ thereby suggesting that the coordination environment of the MOF remains intact after the immobilization of N719. Notably, the ZrBTBD exhibits an additional peak at 2100 cm^–1^, which corresponds to the NCS group of the immobilized N719 molecule.^42^ Moreover, the coordination between ZrBTB and N719 is further verified by the high-resolution O 1s X-ray photoelectron spectroscopy (XPS) data in Figure S2. Here, the unmodified ZrBTB exhibits a noticeable peak at 534.7 eV, which can be attributed to the terminal −OH/OH_2_ groups.^43^ Notably, this peak completely disappears after the immobilization of N719, thereby indicating the coordination of N719 onto the hexa-zirconium clusters of ZrBTB via these terminal −OH/OH_2_ groups during the SALI process. Further, the inductively coupled plasma optical emission spectrometry (ICP-OES) indicates an average loading of 0.2 N719 molecules on each Zr_6_ node of the ZrBTBD. This relatively low loading can be attributed to steric hindrance imposed by the large dye molecules.

Scanning electron microscopy (SEM) images of the N719, ZrBTB, and ZrBTBD are presented in Figure S3. Here, the pristine organic dye exhibits a bulky morphology (Figure S3a), while both the ZrBTB and ZrBTBD exhibit flower-like morphologies composed of stacked nanosheets (Figure S3b,c). Similar morphologies are observed via low-magnification transmission electron microscopy (TEM) in Figure S4, while the high-magnification TEM images in Figure 2d–f reveal that the N719 is composed of large aggregates, while the ZrBTB and ZrBTBD each consist of dispersed 2D sheets. Moreover, no dye aggregates are observed on the ZrBTBD, which clearly indicates the uniform immobilization of N719 molecules on the 2D MOF sheets. Further, the energy dispersive X-ray spectroscopy (EDS) elemental mappings in Figure S5 reveal the uniform spatial distributions of the elements Zr, Ru, O, and C on the stacked ZrBTBD nanosheets. These comprehensive results demonstrate that the N719 dye molecules are uniformly coordinated on the 2D MOF.

### 2.2 Morphology and Microstructure of CNT/MOF Composite Films

This section describes the morphology of the CNT/MOF composite materials. The compositions were adjusted by varying the relative ratios of CNTs and MOFs, resulting in composite systems labeled as C/ZrBTB*x* and C/ZrBTBD*x*, where *x* denotes the weight percentage of MOFs in the composite. In addition, composites containing a fixed 10 wt % MOF were further doped and designated as C/ZrBTB-N*y* and C/ZrBTBD-N*y*, where *y* denotes the N-DMBI concentration (10^–2^ mg mL^–1^) in the composite solution. More detailed experimental procedures and formulations can be found in the Supporting Information.

The surface morphologies of pristine CNTs and their composites with ZrBTB and ZrBTBD (each at 10 wt %) were systematically investigated using scanning electron microscopy (SEM), Figure 2g–i, with additional images provided in Figures S6–S11. The dispersion state of CNTs can serve as an indicator of the interfacial interaction strength between CNTs and MOFs. As shown in Figure 2g, the pristine CNTs tend to form large bundles due to strong π-π stacking and van der Waals interactions. The corresponding statistical histogram (Figure S6a) reveals an average bundle diameter of 97.6 ± 59.6 nm, thereby indicating a significant CNT agglomeration.^44^ Meanwhile, upon incorporation of either ZrBTB or ZrBTBD, the 2D MOF nanosheets are clearly seen to be embedded within the CNT network, while the surface characteristics of these MOFs significantly influence the dispersion of CNTs, thereby altering the morphology of each composite. Nevertheless, as shown in Figure 2h, the incorporation of ZrBTB still contributes to improved CNT dispersion compared to the pristine CNTs, as evidenced by the narrower bundle size distribution (Figure S6b), with an average diameter of 37.8  ±  25.4 nm. In contrast, the C/ZrBTBD10 composite (Figure 2i) exhibits a more pronounced reduction in CNT bundle size. This improvement in dispersion can be attributed to the presence of N719 dye molecules immobilized on the surface of ZrBTBD. Moreover, the bipyridine and conjugated aromatic moieties in N719 facilitate strong π–π stacking interactions with the π-conjugated surfaces of CNTs, which enhance the interfacial compatibility between the MOF and CNTs. Scanning transmission electron microscope (STEM) dark-field images at different magnifications (Figure S25) reveal that CNTs are entangled within the ZrBTB, suggesting a strong interaction between them. Furthermore, C/ZrBTBD exhibits a higher degree of entanglement and interfacial contact compared to C/ZrBTB. This indicates that the immobilization of N719 dye molecules on ZrBTB enhances the interaction between the framework and CNTs, thereby facilitating CNT dispersion. As a result, the composite exhibits smaller and more uniformly distributed MOF domains. Hence, the corresponding histogram in Figure S6c shows an average CNT bundle diameter of 20.3 ± 7.9 nm, thereby indicating the most uniform dispersion and finest CNT network among the various samples. Figures S7 also present SEM images of C/ZrBTB-N5 and C/ZrBTBD-N5 after N-DMBI doping, revealing that the introduction of N-DMBI has minimal impact on the composite morphology.

SEM images of CNT/MOF composites with varying MOF loadings are shown in Figure S8. When the MOF content is 5 wt % (Figure S8a,d), the surface morphology is similar to the 10 wt % samples. However, when the MOF content exceeds 20 wt % (Figure S8b,c,e,f), significant self-aggregation of the MOF nanosheets is observed, leading to the reaggregation of their original flower-like structures and localized CNT bundle formation. This aggregation introduces pores and defects, which are detrimental to charge transport and may degrade overall electrical performance.^45^ In addition, low-magnification SEM images (Figures S9–S11) provide further evidence that this morphological disruption is widespread across the composite films at higher MOF loadings.

To evaluate the surface roughness of the composite films and assess its potential effect on PT conversion, the atomic force microscopy (AFM) results are presented in Figure S12. Here, the pristine CNT sample exhibits a root-mean-square roughness (*R*_RMS_) of 32.3 nm (Figure S12a), while the C/ZrBTBD10 composite exhibits a significantly increased *R*_RMS_ of 44.1 nm (Figure S12b). These results indicate that the ZrBTBD effectively enhances the surface roughness of the composite film. Such morphological changes are expected to enhance the PT conversion efficiency by promoting increased light scattering and trapping, thereby improving the surface light absorption.^46^ By contrast, the rough surface morphology of the C/ZrBTB10 composite exceeded the resolution of AFM scanning, so that no distinct nanosheet morphology could be observed. This is likely due to the larger sheet size, as observed in the corresponding SEM image (Figure 2h). Similarly, reliable AFM measurements could not be obtained at MOF contents of 20 wt % or more due to highly irregular surface features, as observed in the corresponding SEM images (Figure S8b,c,e,f). This highlights the importance of controlling both the dispersion and loading of MOF additives to maintain a uniform surface morphology.

The structural integrity of the MOF after composite formation is verified by the grazing-incidence X-ray diffraction (GIXRD) patterns of the pristine CNTs, the pristine MOFs, and the various CNT/MOF composites in Figures S13 and S14. Thus, the pristine CNTs exhibit sharp diffraction peaks in the *q* range of 2.0–2.7 Å^–1^ (Figure S13a), while both the ZrBTB (Figure S13b) and ZrBTBD (Figure S14a) display characteristic 2D layered MOF peaks in the lower *q* range of 0.3–0.7 Å^–1^, along with ring-like patterns indicating polycrystallinity and random sheet orientations. This is consistent with the XRD results in Figure 2a above. Importantly, the C/ZrBTB and C/ZrBTBD composites each exhibit clear diffraction features corresponding to the presence of CNTs and MOFs, thereby confirming the structural preservation of the MOFs within the composite film. As expected, the intensity of the MOF-related peaks increase with MOF content. Moreover, the structural stability and crystallinity of the MOFs within the composites are further verified by the 1D out-of-plane (*q*_*z*_) profiles in Figure S15.

In summary, both the surface chemistry and loading amounts of MOFs critically govern the dispersion, microstructure, and interfacial architecture of the CNT/MOF composites. The introduction of the surface-functional dye N719 significantly enhances CNT dispersion and interfacial compatibility through strong π–π interactions. Meanwhile, moderate MOF incorporation promotes improved CNT dispersion and surface roughness, which are favorable for enhanced PT performance, whereas excessive MOF loading tends to cause aggregation and the formation of structural defects. Further, the above GIXRD analysis confirmed that the MOF frameworks remain structurally stable within the composite matrices. These results underscore the significance of rational interfacial engineering and additive optimization in the development of high-performance CNT/MOF composite films for PTE applications.

### 2.3 Spectroscopic Analysis of CNT/MOF and N-Doped CNT/MOF Composites

To clarify the interactions between CNTs, MOFs, and the N-DMBI dopant, along with their influence on the thermoelectric (TE) behavior, the results of Raman spectroscopy and ultraviolet photoelectron spectroscopy (UPS) are presented in Figures 3 and S16. First, the Raman spectra of the pristine CNTs and the various C/ZrBTB composites are presented in Figure 3a, while those of the various C/ZrBTBD composites are presented in Figure 3b. Thus, the Raman spectrum of the pristine CNTs exhibits characteristic D and G bands at approximately 1350 cm^–1^ and 1593 cm^–1^, corresponding to disordered sp^2^-hybridized carbon (defects or edge sites) and the in-plane stretching vibrations of the graphene lattice, respectively.^47^ The ratio of intensities between the D and G bands (*I*_D_/*I*_G_) is commonly used to evaluate the degree of structural defects in CNTs. Notably, the addition of MOFs does not significantly change the *I*_D_/*I*_G_ value, which remains at approximately 0.02 across all composites, thereby indicating that the structural integrity of the CNTs remains preserved during composite formation.

**Figure 3:**
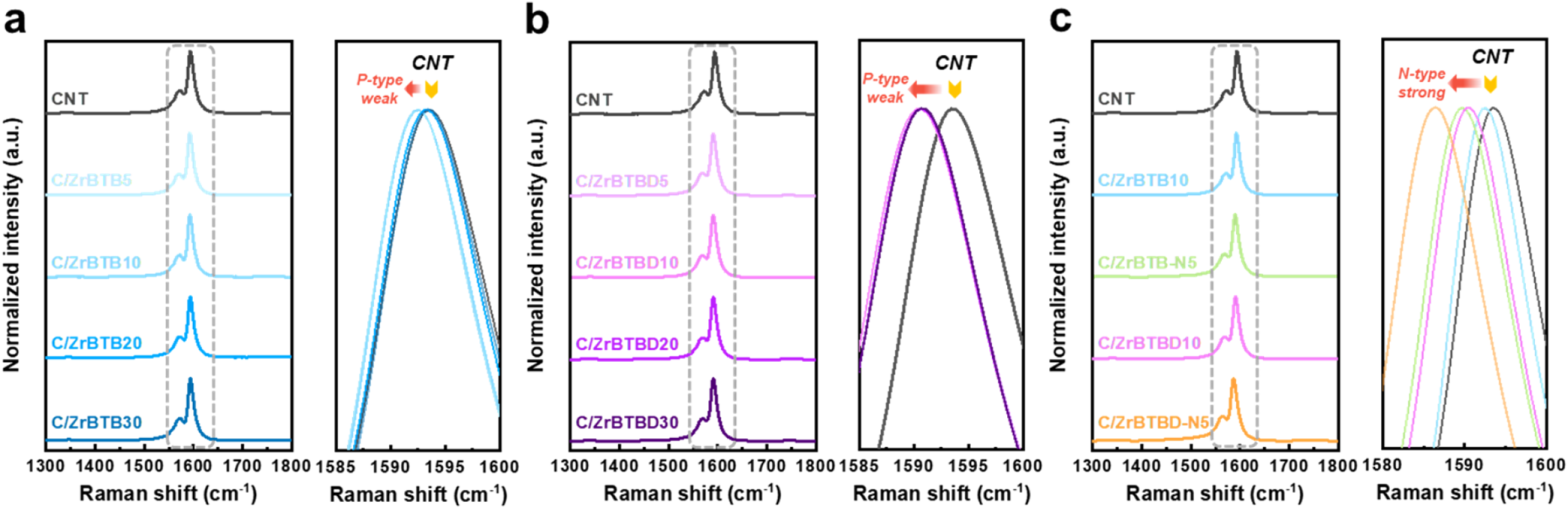
Raman spectra of CNT/MOF composites: (a) C/ZrBTB and (b) C/ZrBTBD with varying MOF content; (c) N-DMBI doped CNT/MOF composites.

The doping level of CNTs was further evaluated by monitoring the shift in the G-band position. CNTs are known to exhibit unintentional p-type doping due to environmental exposure to moisture and oxygen.^48,49^ The corresponding variations are highlighted in the gray-framed regions and their magnified insets (Figure 3a,b). A redshift of the G band generally indicates a reduction in p-type doping or a potential transition toward n-type behavior.^50,51^ In the C/ZrBTB series (Figure 3a), the G-band exhibits only a slight shift from 1593 cm^–1^ to approximately 1592 cm^–1^, thereby suggesting a modest p-type dedoping effect. This behavior may result from the partial shielding of CNTs by the ZrBTB nanosheets,^52^ thereby mitigating, but not fully eliminating, the ambient-induced p-type doping. In contrast, the C/ZrBTBD series (Figure 3b) displays a more pronounced redshift of the G band to around 1590 cm^–1^, indicating a more significant dedoping effect. This can likely be attributed to the presence of N719 dye molecules immobilize on the ZrBTBD surface. This dye contains tetrabutylammonium (TBA^+^) cations, which are known to act as electron donors that can counteract p-type doping in CNTs.^53,54^ Beyond charge compensation, N719 also enhances π–π interactions with CNTs and modulates interfacial carrier concentrations, contributing to more effective p-type doping suppression.

Given that TE modules require both p-type and n-type elements, the organic n-type dopant N-DMBI was employed to invert carrier polarity.^55,56^ To investigate the role of MOF surface chemistry in modulating N-DMBI doping behavior, Raman spectra of C/ZrBTB10 and C/ZrBTBD10 doped with the same N-DMBI concentration (5 × 10^–2^ mg mL^–1^) were compared (Figure 3c). In both cases, the addition of N-DMBI causes a further redshift of the CNT G-band, which is consistent with successful electron injection. However, the shift is more pronounced in the C/ZrBTBD-N5 than in the C/ZrBTB-N5, thereby indicating a higher n-type doping efficiency in the presence of the N719 dye. This is likely facilitated by the pre-existing p-type dedoping effect of the ZrBTBD on the CNTs, which modulates the local electronic environment of the composite to favor the subsequent N-DMBI doping.

To validate these observations, UPS measurements were conducted to determine changes in work function (WF), which correlates with the Fermi level and doping level of the materials.^57^ As shown in Figure S16a, pristine CNTs exhibit a WF of 4.48 eV. Upon MOF incorporation, the WF decreases to 4.40 eV for C/ZrBTB10 (Figure S16b) and to 4.28 eV for C/ZrBTBD10 (Figure S16c), thereby confirming the suppression of p-type doping and a corresponding upward shift of the Fermi level. This trend is consistent with the above-mentioned redshifts in the Raman G-band. Moreover, after N-DMBI doping, the WF exhibits a further decrease to 4.16 eV for the C/ZrBTB-N5 (Figure S16d) and to 3.98 eV for the C/ZrBTBD-N5 (Figure S16e), consistent with effective electron injection. Importantly, under identical doping conditions, C/ZrBTBD-N5 exhibits a significantly lower WF than its ZrBTB-based counterpart, further highlighting the role of immobilized N719 dye on the surface in facilitating and stabilizing n-type doping. This effect is further enhanced by dye modification, indicating additional electron injection. This process effectively neutralizes the holes and suppresses the overall carrier concentration. Consequently, although electrical conductivity decreases due to reduced carrier concentration, the Seebeck coefficient is significantly enhanced, consistent with the inverse relationship between carrier concentration and thermopower. Figure S27 schematically illustrates the energy level alignment and carrier modulation within the CNT/MOF system. The pristine CNTs exhibit p-type characteristics, while composite formation induces a downward shift in work function, suggesting electron transfer from the ZrBTB to the CNTs.

These UPS results provide electronic-level confirmation of the Raman-based assessments and suggest the crucial role of interfacial chemistry in determining the doping response. Taken together, the spectroscopic results establish a clear correlation between MOF surface functionality, interfacial charge modulation, and doping efficacy. The ZrBTBD framework, enriched with surface-bound N719 dye, not only enhances light absorption but also plays a critical role in suppressing unwanted p-type behavior and improving the efficacy of n-type doping. To construct a more holistic understanding of the structure–property relationships in such CNT/MOF-based PTE materials, these molecular insights are further correlated with various TE performance metrics in the following section.

### 2.4 Thermoelectric Performance of CNT/MOF and N-Doped Composite

The PTE effect arises from the synergistic coupling of PT and TE conversion. Therefore, the evaluation of the key TE properties, including the Seebeck coefficient, electrical conductivity, and power factor, is a critical metric for assessing potential of CNT/MOF composites in PTE applications. The TE properties, of pristine CNTs and CNT/MOF composites are shown in Figure 4 and Table S1. As shown in Figure 4a, pristine CNTs exhibit a positive Seebeck coefficient of 21.6 ± 0.5 μV K^–1^, which is indicative of p-type behavior. This relatively low value is attributed to excessive hole doping caused by the adsorption of ambient oxygen and moisture.^49^ To mitigate this effect and improve TE performance, the incorporation of 2D MOF nanosheets with tailored surface functionalities has been proposed as an effective strategy.^32^ Although ZrBTB and ZrBTBD share the same framework structure, they exhibit distinct surface functionalities, which are expected to critically influence charge transport and TE behavior in the resulting composites. It can be also observed that the incorporation of either ZrBTB or ZrBTBD into the CNTs leads to an enhancement in the Seebeck coefficient, confirming their dedoping capability. Specifically, the Seebeck coefficient of the C/ZrBTB10 exhibits a moderate increase to 25.1 ± 2.5 μV K^–1^, while that of the C/ZrBTBD10 demonstrates a more pronounced increase to 62.9 ± 1.6 μV K^–1^. This enhancement is attributed to the immobilized N719 dye on the surface of ZrBTBD, where tetrabutylammonium (TBA^+^) counterions of the N719 regulate the doping level of the composites, thereby improving their TE performance, as supported by Raman and UPS measurements. In addition, aggregated CNTs typically exhibit high carrier concentration due to strong π-π interactions. ZrBTBD possesses a strong interaction with CNTs, effectively dispersing them and creating abundant heterogeneous interfaces. These interfaces modulate the carrier concentration to an optimal level. Consequently, although electrical conductivity is reduced, the Seebeck coefficient improves significantly, leading to an optimized power factor.

**Figure 4:**
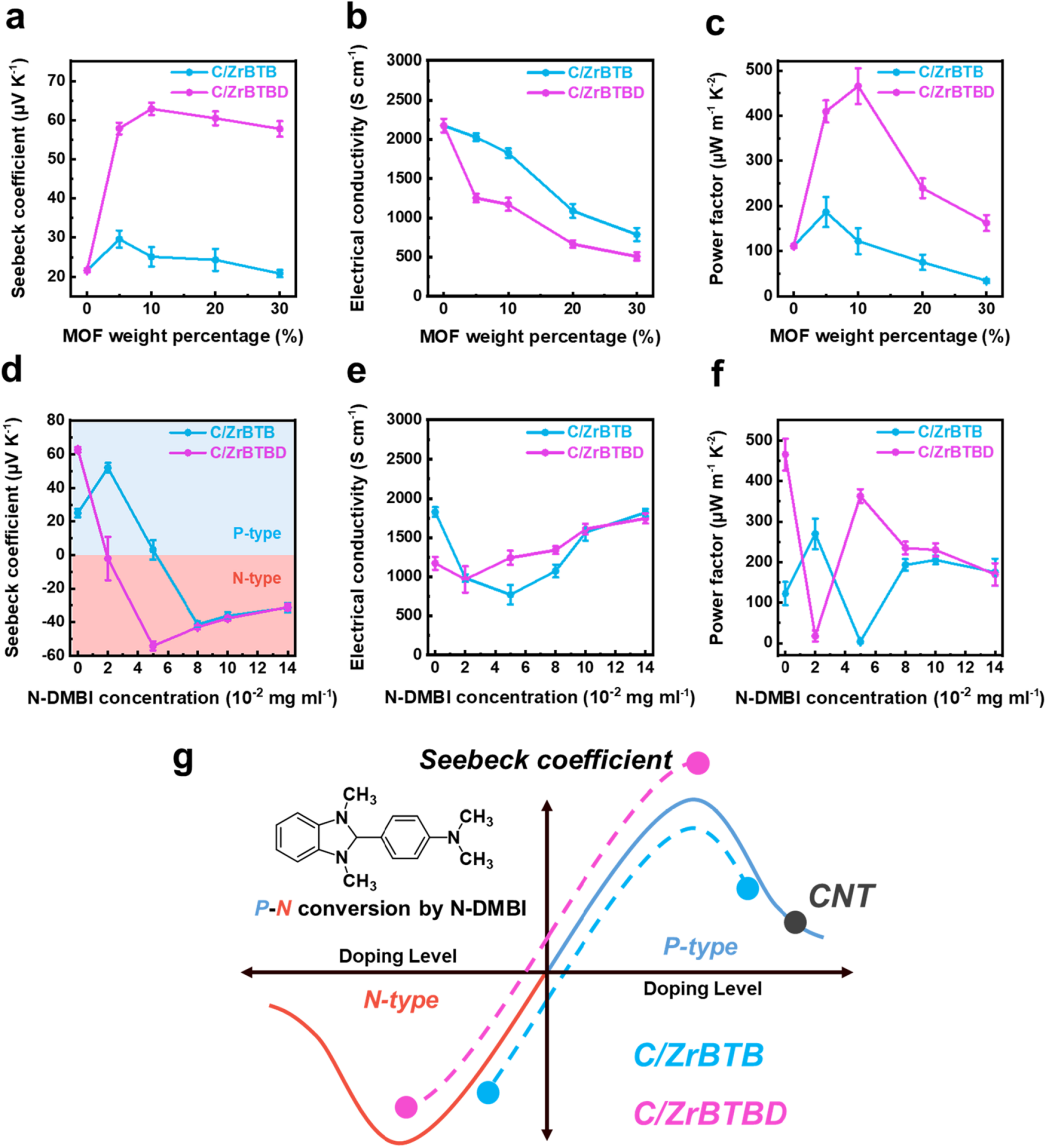
Thermoelectric properties of C/ZrBTB and C/ZrBTBD composites with varying MOF weight percentages: (a) Seebeck coefficient, (b) electrical conductivity, and (c) power factor. Thermoelectric properties of C/ZrBTB and C/ZrBTBD composites doped with different amounts of N-DMBI: (d) Seebeck coefficient, (e) electrical conductivity, and (f) power factor. (g) Schematic illustration showing the correlation between doping level and the Seebeck coefficient of CNT/MOF composites.

To further elucidate the modulation effect of N719 dye, a control experiment was conducted in which pristine CNTs were physically mixed with N719 dye. This resulted in a Seebeck coefficient of −29.6 μV K^–1^, indicative of n-type behavior (Table S2). However, the high standard deviation (±13.5 μV K^–1^) suggests poor doping stability due to dye self-aggregation and lack of interfacial control. In comparison, MOF-immobilized N719 imparts more stable and uniform doping due to controlled interfacial interaction and stable doping level modulation.

As shown in Figure 4b, shows that the electrical conductivity of the composites decreased significantly with increasing MOF content. Pristine CNTs exhibited high conductivity (2176.9 ± 86.8 S cm^–1^), whereas both ZrBTB and ZrBTBD are highly insulating (5.3 × 10^–12^ and 1.4 × 10^–11^ S cm^–1^, respectively; Figure S17). Consequently, the incorporation of MOF domains disrupts the conductive percolation network and introduces interfacial barriers, which impede carrier transport. However, such structural heterogeneity may enhance phonon scattering and reduce thermal conductivity, which can be a desirable trade-off in TE materials. It should be noted that the C/ZrBTB series consistently exhibits higher electrical conductivity than the C/ZrBTBD series, likely due to its stronger susceptibility to ambient p-type doping. For example, C/ZrBTB10 exhibits an electrical conductivity of 1828.1 ± 59.9 S cm^–1^, whereas C/ZrBTBD10 reaches only 1172.0 ± 82.7 S cm^–1^. Notably, increasing the MOF content beyond 10 wt % results in a decline in both Seebeck coefficient and electrical conductivity (Figure 4a,b), due to morphological inhomogeneities and MOF aggregation observed in SEM images (Figure S8b,c,e,f). Thus, at an MOF content of 30 wt %, the Seebeck coefficients of C/ZrBTB30 and C/ZrBTBD30 decrease to 20.8 ± 0.9 and 57.8 ± 2.0 μV K^–1^, while electrical conductivity decreases to 788.0 ± 85.1 and 508.0 ± 53.1 S cm^–1^respectively, highlighting the importance of optimized MOF content.

The power factor (*PF* = *S* ^2^σ), presented in Figure 4c. Here, pristine CNTs yield a modest *PF* of 111.2 ± 4.3 μW m^–1^ K^–2^, which is limited by their low Seebeck coefficient due to excessive carrier doping. Meanwhile, the introduction of the 2D MOFs enhances the Seebeck coefficient by modulating the doping level while maintaining the intrinsically high electrical conductivity of the CNTs, thus leading to improved *PF* values. While C/ZrBTB10 composite exhibits a *PF* of 186.5 ± 33.3 μW m^–1^ K^–2^, the enhancement is still limited by relatively weak dedoping effect. In contrast, the C/ZrBTBD10 composite achieves an excellent *PF* of 465.7 ± 39.5 μW m^–1^ K^–2^, approximately 4-fold higher than pristine CNTs, primarily due to its significantly enhanced Seebeck coefficient. This improvement is driven by the synergistic effects of N719 dye interfacial engineering and charge modulation upon doping.

The Seebeck coefficients obtained after N-DMBI doping for the various composites are shown in Figure 4d. Interestingly, C/ZrBTBD-N2 exhibits a n-type Seebeck coefficient of −2.1  ±  12.9 μV K^–1^, while C/ZrBTB-N2 retain an unexpectedly enhanced Seebeck coefficient of 52.1  ±  2.9 μV K^–1^, increased from 25.1  ±  2.5 μV K^–1^. This phenomenon is attributed to an initial overdoping effect of C/ZrBTB10 composite, in which at low N-DMBI concentrations, partial dedoping reduces hole concentration but does not induce a polarity switch. As schematically illustrated in Figure 4g, the C/ZrBTBD10 composite lies closer to the p–n transition threshold, enabling a polarity flip at lower dopant concentrations. At higher N-DMBI levels (above 8 × 10^–2^ mg mL^–1^), both the C/ZrBTB and C/ZrBTBD systems converge in Seebeck coefficients, indicating a saturation of accessible doping sites and diminished influence of MOF surface chemistry.

The electrical conductivity of doped composites (Figure 4e) shows different trends for the two series. In C/ZrBTB series, initial N-DMBI doping reduces electrical conductivity due to electron–hole recombination, after which the conductivity recovered at higher dopant concentrations as electron injection dominates. For instance, electrical conductivity in C/ZrBTB-N14 recovers to 1819.5 ± 46.8 S cm^–1^. In contrast, C/ZrBTBD composites show a monotonic increase in electrical conductivity, from 965.5 ± 168.5 S cm^–1^ (C/ZrBTBD-N2) to 1745.0 ± 64.1 S cm^–1^ (C/ZrBTBD-N14), reflecting more efficient and progressive doping. Consequently, the corresponding *PF* (Figure 4f) reach maxima of 183.3 μW m^–1^ K^–2^ for C/ZrBTB-N8 and 363.1 μW m^–1^ K^–2^ for C/ZrBTBD-N5. Overall, the C/ZrBTBD composites demonstrate excellent TE performance in both p- and n-type regions. Specifically, C/ZrBTBD10 achieves a record high Seebeck coefficient of 62.9 μV K^–1^ and a *PF* of 465.7 μW m^–1^ K^–2^, while C/ZrBTBD-N5 reaches a Seebeck coefficient and *PF* of −54.2 μV K^–1^ and 363.1 μW m^–1^ K^–2^, respectively. To the best of our knowledge, these values represent the highest reported Seebeck coefficients and *PF*s for CNT/MOF composites to date.^45,58–61^ A comparative summary is provided in Table S3, and a visual benchmark is shown in Figure S18, which further highlights the significance of these results in advancing high-performance composite TE materials. As shown in Figure S26, the electrical conductivity of C/ZrBTBD-N5 remains stable for up to 48 h under ambient conditions, while optimal n-type thermoelectric performance is maintained for approximately 24 h before gradual N-DMBI dedoping occurs. We anticipate that implementing standard encapsulation technologies will effectively suppress this ambient-induced dedoping and significantly prolong long-term stability.

To comprehensively assess the TE performance, thermal conductivities (κ) of optimized composite films are systematically examined in Figure 5. Thermal conductivity comprises electronic (κ_e_) and lattice (κ_l_) contributions. κ_e_ can be estimated using the Wiedemann–Franz law, whereas κ_l_ is governed by phonon transport.^62^ Given their high porosity and insulating nature, MOFs primarily suppress κ_l_, thereby lowering the thermal conductivity of composite films. As shown in Figure 5a, pristine CNT film exhibits a thermal conductivity of 41.5 W m^–1^ K^–1^, and a significant reduction is observed upon the incorporation of MOFs. Specifically, the C/ZrBTB10 and C/ZrBTBD10 have thermal conductivity values of 4.55 and 3.32 W m^–1^ K^–1^, respectively. This difference can be attributed to variations in both κ_e_ and κ_l_. The relative κ_e_ contributions can be deduced from the electrical conductivity data in Figure 4b, where the C/ZrBTB10 exhibits a higher average electrical conductivity of 1828.1 S cm^–1^, compared to 1172 S cm^–1^ for the C/ZrBTBD10, thereby indicating that a greater κ_e_ contribution in the former sample. Further, the SEM images in Figure 2h,i reveal that the ZrBTBD establishes more intimate contact with the CNTs, thus leading to a greater density of heterogeneous interfaces. These interfaces serve as phonon scattering cites, effectively suppressing κ_l_ and contributing to the lower thermal conductivity of the C/ZrBTBD10.

**Figure 5:**
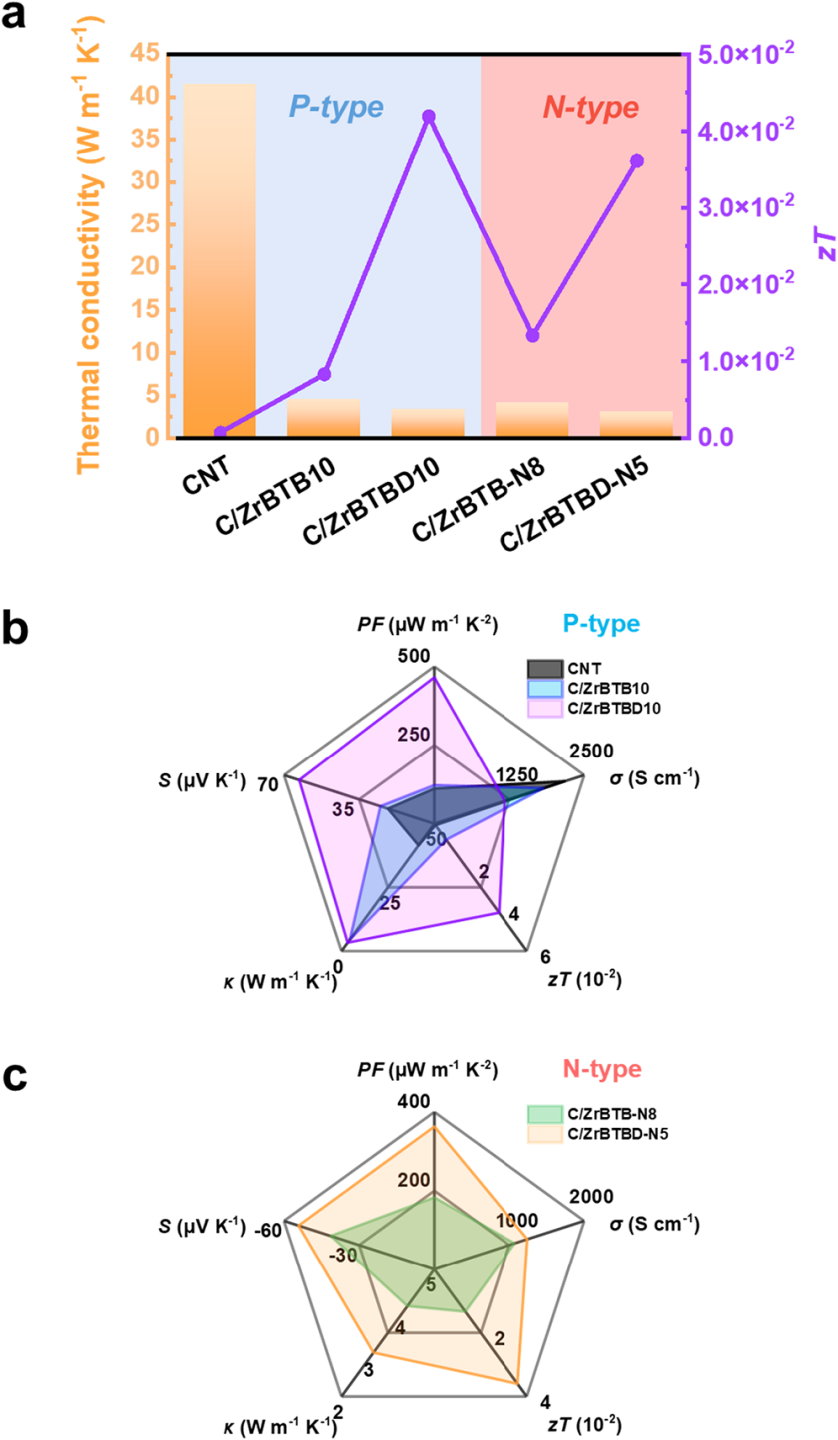
(a) Thermal conductivity and *zT* values of pristine CNT, C/ZrBTB10, C/ZrBTBD10, C/ZrBTB-N8, and C/ZrBTBD-N5. Radar chart comparison of key thermoelectric parameters (*PF*, σ, *S*, κ, and *zT*) for (b) P-type and (c) N-type composites.

The *zT* at 303 K is calculated (Figure 5a). Pristine CNT film exhibits a *zT* of 7.3 × 10^–4^, whereas C/ZrBTB10, and C/ZrBTBD10 achieve significantly enhanced *zT* of 7.6 × 10^–3^ and 4.2 × 10^–2^, respectively, representing 10-fold and 57-fold improvements over the pristine CNT. These results clearly demonstrate the effectiveness of MOF incorporation and surface functionalization in enhancing TE performance. For n-type composites, N-DMBI doping further reduces κ via interface-induced phonon scattering,^63^ yielding κ values of 4.13  W m^–1^ K^–1^ (C/ZrBTB-N8) and 3.03 W m^–1^ K^–1^ (C/ZrBTBD-N5), and corresponding *zT* of 1.3 × 10^–2^ and 3.6 × 10^–2^. Comparative overviews of the p-type and n-type performances of the composites are provided by the radar plots in Figure 5b,c, respectively, which capture the interrelationship between Seebeck coefficient, electrical conductivity, thermal conductivity, and overall *zT*. These visualizations clearly demonstrate that MOF surface modification not only improves the *PF* but also increases the density of phonon-scattering interfaces, thus reducing thermal conductivity and enhancing the overall TE performance (*zT*).

### 2.5 Photothermal Properties of CNT/MOF Composites

To evaluate the photothermal (PT) conversion properties of the pristine CNTs and the C/ZrBTB10, and C/ZrBTBD10 composites, the changes in surface temperature of the thin film samples were measured under controlled illumination by using an infrared (IR) thermal imaging camera in conjunction with a custom-built experimental setup. A solar simulator was used as the irradiation source, and the detailed experimental parameters are provided in the Section 4. Three samples exhibiting superior TE performance were selected for comparison: pristine CNT, C/ZrBTB10, and C/ZrBTBD10. Under illumination at 100 mW cm^–2^, the IR thermal images of these materials are shown in Figure 6a, where the black crosshair indicates the temperature measurement point, and the corresponding temperature–time profiles are presented in Figure 6b. Notably, C/ZrBTBD10 exhibits the most efficient PT conversion, reaching a maximum temperature of approximately 51.2 °C after 180 s of irradiation. In comparison, C/ZrBTB10 and pristine CNT reach about 47.5 °C and 47.3 °C, respectively, indicating that the incorporation of ZrBTB contributes only marginally to overall light-to-heat conversion. In addition, the effect of the N719 dye on the optical absorption behavior of the MOF is elucidated by the UV–vis absorption spectra in Figure 6c. Here, the pristine ZrBTB displays an absorption peak only at around 290 nm, thereby suggesting minimal light absorption in the visible region. By contrast, ZrBTBD exhibits a broad and intense absorption band spanning 350–700 nm, demonstrating significantly enhanced visible-light harvesting and improved photothermal conversion. This enhancement is attributed to the presence of N719 dye molecules on the MOF surface, which strongly absorb in the visible region and promote efficient light-to-heat conversion.^64^ Using a energy-balance method, the C/ZrBTBD10 composite demonstrated a competitive photothermal conversion efficiency of 34.2%, with its moderate operating temperature (∼51 °C) resulting from rapid heat exchange inherent to the thin-film device architecture (Table S4).

**Figure 6:**
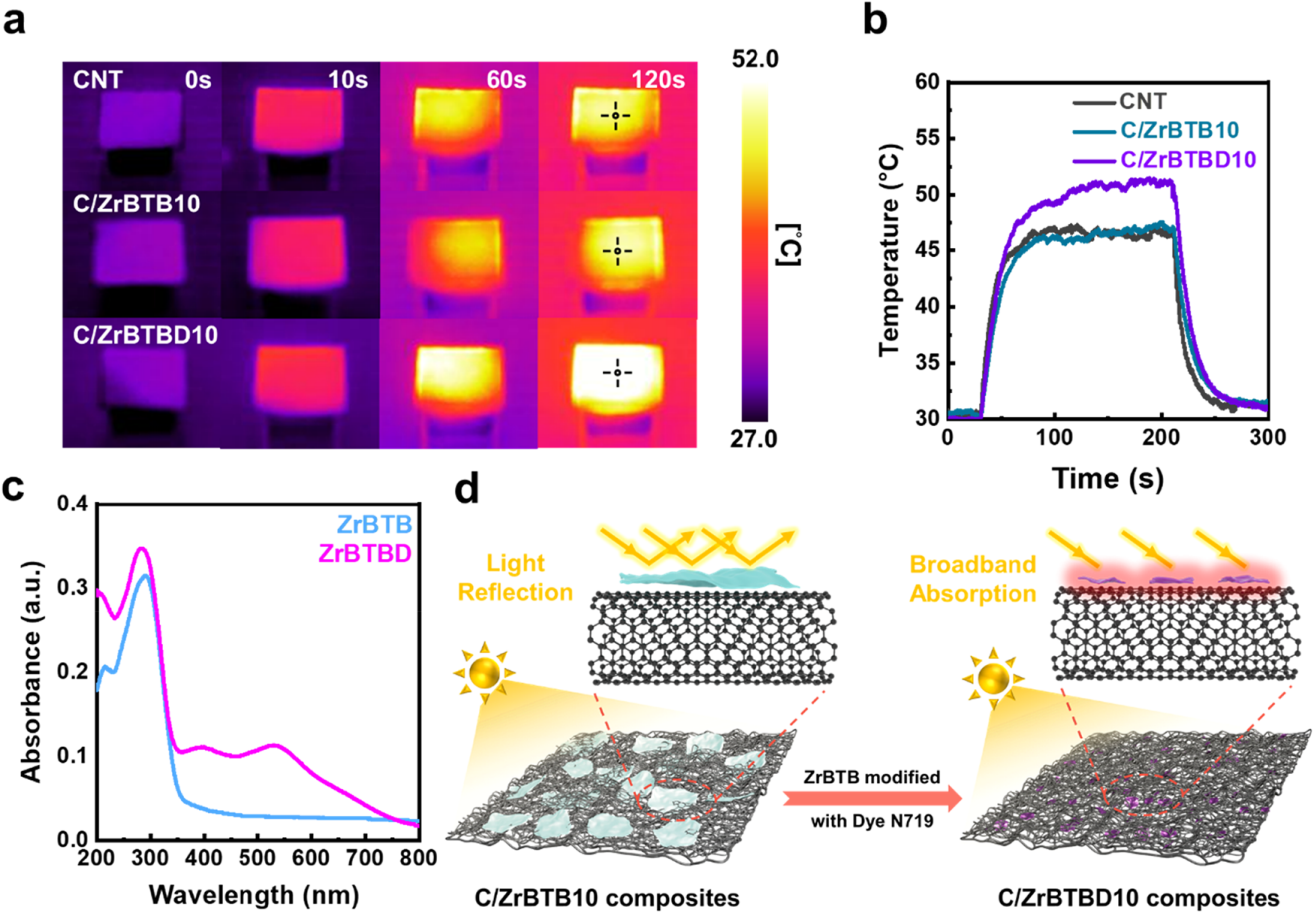
(a) Infrared thermal images of CNT and CNT/MOF composites under solar simulator illumination for different durations. (b) Time-dependent temperature of CNT and CNT/MOF composites. (c) UV–vis spectra of ZrBTB and ZrBTB-D. (d) Schematic of light-harvesting mechanism in CNT/MOF composites.

To quantify the heating kinetics, the thermal response times (defined as the time taken to reach 90% of the steady-state temperature) of the pristine CNTs, the C/ZrBTB10, and the C/ZrBTBD10 were measured, yielding values of 16.2 s, 24.4 s, and 27.5 s, respectively. The observed increase in response time upon MOF incorporation is attributed to the reduced thermal conductivity of the composite, which is a result of the intrinsic insulating nature of the MOFs and the formation of phonon-scattering interfaces at the CNT/MOF boundaries. Although the MOFs delay heat propagation, they also promote heat retention and enable higher final surface temperatures by limiting dissipation. In addition, the morphological differences between the two composites may contribute to their distinct PT behavior. As illustrated schematically in Figure 6d and observed via SEM (Figure 2h), ZrBTB tends to aggregate into large sheet-like domains within the composite film, and these can potentially reflect the incident light and reducing absorption. In contrast, ZrBTBD exhibits finer fragmentation and stronger dispersion within the CNT network (Figure 2i), thereby facilitating light penetration and enhancing internal scattering, which contributes to greater heat accumulation throughout the film matrix. The complete IR images and corresponding temperature–time profiles under varied light intensities (100, 80, and 60 mW cm^–2^) are provided in Figures S19–S23. These data collectively demonstrate the scalability and robustness of the PT conversion behavior across different light intensities.

The PT behavior of N-DMBI-doped samples was also investigated. Since N-DMBI predominantly absorbs in the ultraviolet range,^65^ and that solar-driven PT conversion is mainly driven by visible and near-infrared light, the dopant’s contribution to light–heat conversion is negligible. As shown in Figures S22 and S23d, the PT performance of C/ZrBTBD-N5 remains nearly identical to that of its undoped counterpart, confirming that the primary enhancement in PT efficiency originates from the functionalization with N719 dye rather than the dopant.

### 2.6 Demonstration of a Prototype CNT/MOF Photothermoelectric Generator

To validate the PTE performance of the developed composite materials, a prototype PTE generator (PTEG) was fabricated using the best-performing p-type and n-type materials, namely, the C/ZrBTBD10 and C/ZrBTBD-N5, respectively. A schematic illustration and a photographic image of the fabricated device, which consists of five pairs of p-n TE legs connected in series, are presented in Figure 7a,b, respectively. The detailed fabrication procedures and measurement protocols are provided in the Supporting Information. As shown in Figure 7c, the output voltage of the PTEG increases proportionally with light intensity. Under simulated solar irradiation at 100 mW cm^–2^, the device generates an open-circuit voltage (*V*_oc_) of 12.3 mV. When the light intensity is decreased to 80 and 60 mW cm^–2^, the *V*_oc_ correspondingly decreases to 9.6 and 7.3 mV, respectively. This trend indicates that a higher photon flux induces a greater temperature gradient across the hot and cold ends, thereby resulting in increased voltage output. Further, the thermal stability and cycling durability of the device under continuous light exposure and light on/off switching are demonstrated in Figure 7d,7e. These results reveal that the low thermal conductivity of the C/ZrBTBD10 enables effective maintenance of the temperature gradient during prolonged irradiation. However, a slight decrease in peak *V*_oc_ is observed with successive illumination cycles, which is likely due to heat accumulation and insufficient heat dissipation. This effect gradually raises the background temperature under repeated illumination, thereby reducing the temperature difference achievable within each cycle.

**Figure 7:**
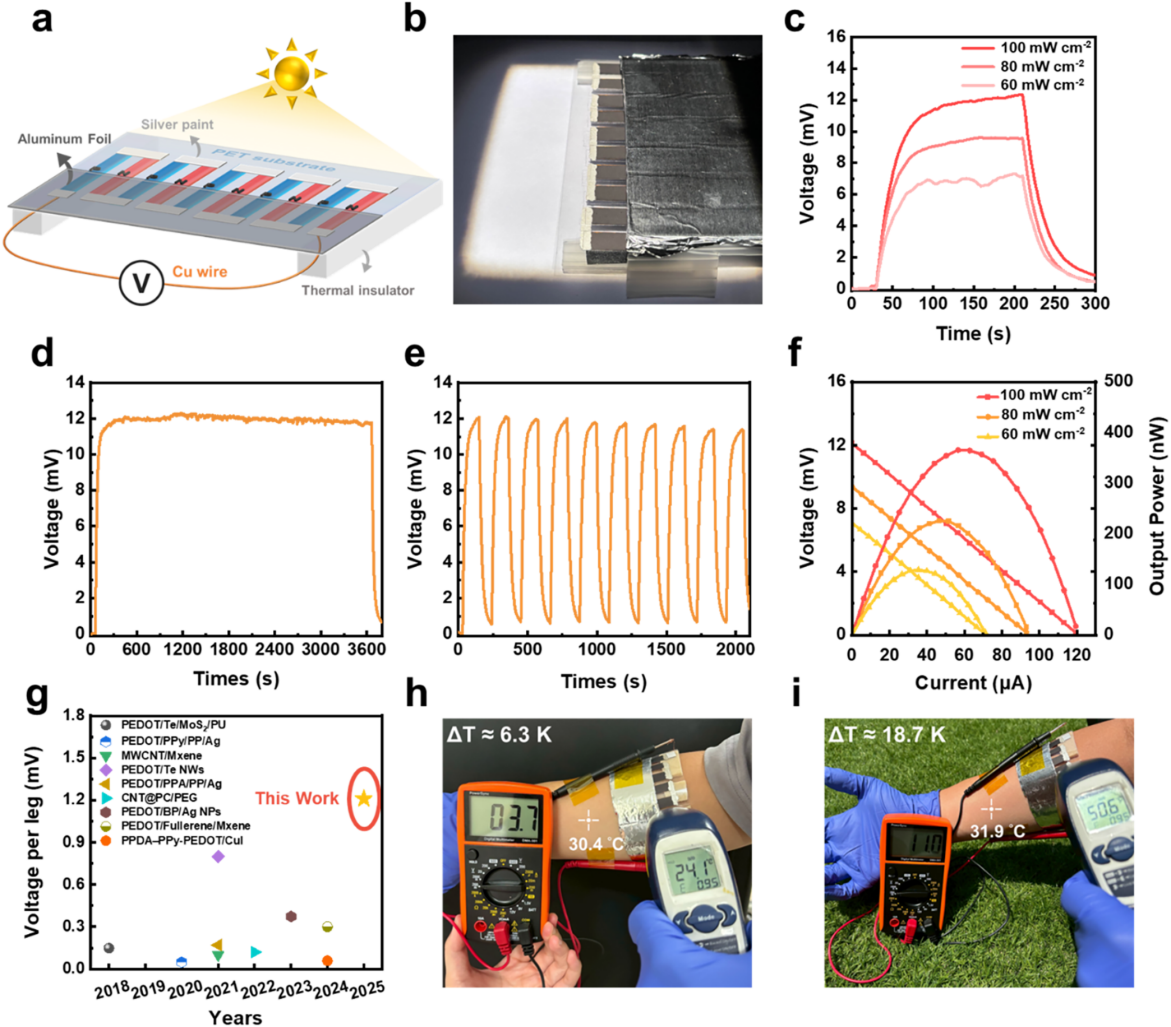
(a) Schematic illustration of the PTE device measurement setup. (b) Optical image of the PTE device. (c) Time-dependent output voltage under 100, 80, and 60 mW cm^–2^ light intensities. (d) Long-term stability test and (e) cycling test of output voltage under 100 mW cm^–2^. (f) Output voltage (left axis) and power (right axis) as a function of current at different light intensities. (g) Comparison of single-leg output voltage under 1 sun for organic–inorganic hybrid material-based homogeneous PTE devices in this work and previous reports. Wearable PTEG device performance under (h) indoor and (i) outdoor environments.

The output voltage and corresponding power output of the PTEG device under various light intensities are presented in Figure 7f. After 5 min of continuous irradiation to ensure thermal equilibrium, the device generates a *V*_oc_ of 12.3 mV and a maximum power output (*P*_max_) of 365.4 nW under 100 mW cm^–2^. At reduced light intensities, the output performance also decreases, with *V*_oc_ and *P*_max_ values of 9.4 mV and 225.3 nW, respectively, at 80 mW cm^–2^, and 7.1 mV and 128.65 nW, respectively, at 60 mW cm^–2^. These results demonstrate the device’s high PTE conversion efficiency and strong responsiveness to varying light intensities. To benchmark device performance, the *V*_oc_ was normalized per TE leg and compared with previously reported organic–inorganic hybrid homogeneous PTE systems (Figure 7g). Remarkably, our PTEG exhibited the highest output voltage per leg among surveyed literature,^4,66–72^ highlighting the superior performance of our CNT/MOF composite system and its promising potential for integration in wearable electronics. Based on the measured *V*_oc_ and *P*_max_, the internal resistance of our optimized PTEG is calculated to be approximately 103.5 Ω using the equation *R* = *V*_oc_^2^/(4 *P*_max_). Although the incorporation of MOFs leads to increased internal resistance due to reduced electrical conductivity, this negative impact is significantly outweighed by the squared contribution of the enhanced Seebeck coefficient to the power factor and the suppressed thermal conductivity, which establishes a larger temperature gradient to boost voltage output and ensures superior overall power performance compared to pristine CNT devices.

The practical applicability of the PTEG as a flexible and wearable device is demonstrated by the mechanical bending test results shown in Figure S24a,b, where the structural integrity of the device is retained under various bending radius. Moreover, negligible changes in resistance are observed during static bending (Figure S24c), thereby indicating excellent mechanical compliance. Furthermore, the results of a 1000-cycle bending durability test at a radius of 8 cm (approximating the curvature of a human forearm) are presented in Figure S24d. Here, the resistance remains nearly unchanged throughout, thereby confirming the mechanical durability and operational reliability of the device for wearable applications. Finally, to assess real-world applicability, the PTEG was mounted on a human forearm and tested under both indoor and outdoor conditions. To establish an effective temperature gradient, the nonilluminated side was covered with aluminum foil, while a foam layer was applied to the illuminated region to provide insulation and prevent skin burns. Due to the difference in environmental illumination, the hot–cold side configuration varied between indoor and outdoor settings. Under indoor ambient light (Figure 7h), the skin-contact region reached 30.4 °C, while the shaded cold side remained at 24.1 °C, generating a *V*_oc_ of 3.7 mV. Under direct noon sunlight outdoors (Figure 7i), the PT material allowed the temperature of the illuminated side to increase to 50.6 °C, while the cold side remained at 31.9 °C, yielding a significantly higher *V*_oc_ of 11 mV. These outputs are consistent with simulated solar conditions and validate the device’s strong potential for practical low-power energy harvesting in wearable PTE application. The flexible MOF/CNT composite can be seamlessly integrated into wearable platforms, such as textiles or skin-mounted devices, to serve as a miniature energy harvester or self-powered sensor by utilizing temperature gradients established under solar irradiation. Moreover, owing to its broadband light absorption and thin-film processability, the composite is also compatible with hybrid integration alongside photovoltaic devices as a supplementary energy-harvesting layer.

## 3 Conclusions

In summary, we developed a series of homogeneous PTE composites by integrating CNTs with N719 dye-modified Zr-MOFs. Postsynthetic modification of ZrBTB with N719 dye yielded ZrBTBD, which enhanced visible-light absorption and enabled energy-level tuning to modulate CNT doping behavior. The resulting p-type C/ZrBTBD10 and n-type C/ZrBTBD-N5 composites exhibited excellent TE performance, with PF of 465.7 and 363.1 μW m^–1^ K^–2^, respectively, representing the highest values reported to date among CNT/MOF materials. The enhancement was further supported by a significant increase in the *zT*, attributed to simultaneous suppression of thermal conductivity via phonon scattering at MOF/CNT interfaces. PT characterization confirmed the beneficial role of N719 functionalization, with composite surface temperatures rising from 47.3 °C to 51.2 °C under 100 mW cm^–2^ illumination, reflecting improved light-to-heat conversion efficiency. A flexible PTE generator (PTEG) assembled from the optimized p-type and n-type composites delivered a *V*_oc_ of 12.3 mV and a *P*_max_ of 365.4 nW under standard illumination. Notably, the voltage per TE leg surpassed all previously reported organic–inorganic hybrid homogeneous PTE systems. The device also demonstrated excellent mechanical durability and operational stability, maintaining consistent electrical performance after 1000 bending cycles and prolonged light exposure. Importantly, this work represents the first successful demonstration of MOF-based composite materials in homogeneous PTE systems, offering a new platform for light-responsive and energy-harvesting devices. The integration of PT and TE functionalities in a single composite system not only simplifies device architecture but also advances the practical application of flexible and wearable power sources for next-generation electronics.

## 4 Experimental Section

### 4.1 Preparation of C/ZrBTB and C/ZrBTBD Composite Solution

To prepare the composite precursors, ZrBTB and ZrBTBD powders were first vacuum-dried at 80 °C for 12 h to eliminate residual moisture and solvents. During formulation, the total solid content in each solution was precisely controlled at 1 mg mL^–1^. The compositions were adjusted by varying the relative ratios of carbon nanotubes (CNTs) and metal–organic frameworks (MOFs), resulting in composite systems labeled C/ZrBTB*x* and C/ZrBTBD*x*, where *x* denotes the weight fraction of MOFs in the composite. As a representative example, the formulation of C/ZrBTB5 involved dispersing 0.15 mg of ZrBTB into 3 mL of DCB, followed by ultrasonication to facilitate uniform dispersion. Higher loadings, such as 0.3 mg (C/ZrBTB10), 0.6 mg (C/ZrBTB20), and 0.9 mg (C/ZrBTB30), along with their ZrBTBD counterparts, were prepared using an analogous protocol. CNTs were then introduced into each dispersion to restore the overall solid content to 1 mg mL^–1^. The final mixtures were mechanically homogenized using a Retsch MM440 ball mill operating at 30 Hz for 15 min. For the preparation of n-type variants, the MOF content was maintained at a constant 10 wt %, while the N-DMBI were varied. These formulations were designated as C/ZrBTB-N*y* and C/ZrBTBD-N*y*, with *y* representing the concentration (10^–2^ mg mL^–1^) of N-DMBI in the final mixture. For instance, to obtain C/ZrBTB-N2, 0.06 mg of N-DMBI was introduced into a 3 mL dispersion of the 10 wt % MOF composites, followed by the same homogenization process. Additional variants such as C/ZrBTB-N5 (0.15 mg), C/ZrBTB-N8 (0.24 mg), C/ZrBTB-N10 (0.3 mg), and C/ZrBTB-N14 (0.42 mg), along with their ZrBTBD analogues, were synthesized using the same approach.

### 4.2 Fabrication of Thermoelectric Film and Photothermal Film

To fabricate composite films, the prepared solution was applied onto cleaned glass substrates (7.5 × 15 mm^2^) using a two-step drop-casting method. Before deposition, the substrates were treated with sequential ultrasonic baths of acetone, isopropanol, and deionized water, followed by ozone plasma treatment to eliminate residual surface contaminants. The cleaned substrates were then preheated on a hot plate at 100 °C to facilitate solvent evaporation and ensure uniform film formation. After preheating, 100 μL of the composite solution was cast onto the substrate in two aliquots and dried at the same temperature. To remove residual solvent and improve the interfacial adhesion between the CNT and the MOF matrix, the dried films were annealed at 190 °C for 10 min. The resulting films had a thickness in the range of approximately 3 to 5 μm. All procedures involving film deposition and thermal treatment were conducted within a nitrogen-filled glovebox to prevent exposure to moisture and oxygen. For the evaluation of photothermal properties, composite films were also prepared on polyimide (PI) substrates measuring 1.5 × 1.5 cm^2^. In these experiments, the casting volume was increased to 200 μL while keeping other processing parameters unchanged.

## Supplementary Material



## Notes

Extended morphological data; supplementary spectroscopic results; and comprehensive device performance characterization (PDF)
