# Supplementary material for: Organic Dye-Modified Two-Dimensional Metal–Organic Framework/Carbon Nanotube Composite Films for Photothermoelectric Applications
Source: ACS Nano. 2026 Apr 8;20(16):12425–39. doi: 10.1021/acsnano.6c00103 (PMC13131043; doi:10.1021/acsnano.6c00103)
Supplement: Supplementary file 1 [file nn6c00103_si_001.pdf]

Supporting Information

Organic Dye-Modified Two-Dimensional Metal–Organic Framework/Carbon Nanotube Composite Films for Photothermoelectric Applications

Cheng-Yuan Lin,[a] Kuan-Chu Wu,[b] Chih-Wei Hsu,[a] Shao-Huan Hong,[a] Chi-Lun Chuang,[b] Te-Jen Hsu,[a] Jhih-Min Lin,[c] Chung-Wei Kung[b], Cheng-Liang Liu[a,d,e]**

[a] Department of Materials Science and Engineering, National Taiwan University, Taipei 10617, Taiwan

[b] Department of Chemical Engineering, National Cheng Kung University, Tainan 701401, Taiwan

[c] National Synchrotron Radiation Research Center, Hsinchu 30076, Taiwan

[d] Institute of Polymer Science and Engineering, National Taiwan University, Taipei 10617, Taiwan

[e] Advanced Research Center for Green Materials Science and Technology, National Taiwan University, Taipei 10617, Taiwan

*E-mail: cwkung@mail.ncku.edu.tw (C.-W. Kung); liucl@ntu.edu.tw (C.-L. Liu)

KEYWORDS. metal–organic framework, photothermoelectric, dye, carbon nanotube, thermoelectric, wearable, composite

Materials. Zirconium (IV) chloride ($ZrCl_4$, 98%, Acros Organics), benzoic acid (BA, 99.5%, Sigma-Aldrich), 1,3,5-tri(4-carboxyphenyl)benzene (H3BTB, 97%, Alfa Aesar), N719 dye (≥98%, Lumtec, Taiwan), N,N-dimethylformamide (DMF, 99.9%, Duksan Pure Chemicals), dimethyl sulfoxide (DMSO, 99%, Duksan Pure Chemicals), hydrochloric acid (HCl, 36.5-38.0%, J.T. Baker), acetone (ECHO Chemical, Taiwan, 98%), deionized water were utilized throughout the study. Single-wall carbon nanotubes (SWCNTs, diameter < 2 nm, length > 5 μm, 85%) were obtained from Tuball™. 1,2-dichlorobenzene (DCB, anhydrous 99%) was obtained from Sigma-Aldrich. PELCO® conductive silver paint was obtained from TED PELLA, Inc. All chemicals were used as received without purification.

Characterization. The crystal structures of the MOF powders were characterized by X-ray diffraction (XRD) using a SmartLab diffractometer (Rigaku). Specific surface areas were evaluated via nitrogen adsorption–desorption isotherms on a 3Flex surface characterization analyzer (Micromeritics). Fourier-transform infrared (FTIR) spectra were collected using a Nicolet 6700 spectrometer (Thermo Fisher Scientific), while ultraviolet–visible (UV–Vis) absorption spectra were recorded with a UV2600 spectrophotometer (Shimadzu). Elemental compositions were determined by inductively coupled plasma optical emission spectroscopy (ICP-OES) using a JY 2000–2 instrument (Horiba Scientific). Electrical conductivity measurements were conducted with a CHI1205C electrochemical workstation (CH Instruments Inc.). Morphological features were examined by scanning electron microscopy (SEM) using a Hitachi SU-8010 system. Transmission electron microscopy (TEM) images were obtained with a Hitachi H-7500 microscope, and energy-dispersive X-ray spectroscopy (EDS) analysis was carried out on a JEOL JEM-1400 Flash instrument. X-ray photoelectron spectroscopy (XPS) was conducted using a Theta Probe system (Thermo Scientific) to investigate the surface elemental composition and chemical states of the samples.

For composite film, the thickness of the composite films was measured using a KLA-Tencor D300 profilometer. To improve accuracy, five different positions on each sample were

measured, and the average value was used as the representative thickness. Surface morphology of composites was analyzed by field-emission scanning electron microscopy (FE-SEM; JEOL JSM-7600F) and atomic force microscopy (AFM; Hitachi AFM5100N), using an SI-DF3PS probe with a resonance frequency of 70 kHz and a force constant of 2.0 N m^{-1}. Grazing-incidence X-ray diffraction (GIXRD) was carried out at the TPS 25A beamline of the National Synchrotron Radiation Research Center (NSRRC), Taiwan, to investigate the crystallographic structure of the films. Raman spectroscopy was performed using a Jobin Yvon LabRAM HR800 UV spectrometer equipped with a 532 nm Nd:YAG laser as the excitation source, calibrated using a silicon (Si) reference peak at 520.71 cm^{-1}. Ultraviolet photoelectron spectroscopy (UPS) was conducted with a PHI 5000 Versa Probe III system (ULVAC-PHI Inc.) under ultra-high vacuum (6.7×10^{-8} Pa), using He I radiation (21.22 eV) as the excitation source.

Synthesis of ZrBTB and ZrBTBD. The synthesis of ZrBTB is based on our previous work, with benzoic acid as the modulator during the MOF growth, followed by the removal of coordinated benzoate ligands to obtain accessible terminal –OH/OH_2 groups.[1] To further coordinate N719 on the 2D sheets of ZrBTB, 91 mg of N719 was dissolved in 14 mL of N,N-dimethylformamide (DMF), corresponding to two equivalents of nodes of the ZrBTB added later. The mixture was sealed in a glass vial and ultrasonicated for 10 min to obtain a homogeneous solution. Subsequently, 40 mg of ZrBTB was added into the dye solution, and the resulting mixture was further sonicated for another 10 min to fully disperse MOF sheets. The suspension was then heated in an oil bath at 60 °C under constant stirring at 300 rpm for 18 h. After the reaction, the solid product was subjected to successive washing processes through centrifugation. The MOF solid was first washed by 14 mL of DMF seven times; a fully colorless supernatant was obtained after the last round of centrifugation. Solvent exchange was then performed by immersing the MOF solid in 14 mL of fresh acetone three times over the course of overnight, and the resulting solid was placed in a vacuum oven at 60 °C overnight. The final product exhibiting a color in dark purple was designated as "ZrBTBD".

Pellets of ZrBTB and ZrBTBD were prepared, and the electrical conductivity of each MOF was estimated by utilizing the two-probe method. See experimental details from our previous work.[13] The cross-section area and thickness of pellets of both materials are 0.3848 cm^2 and 0.028 cm, respectively, and the obtained current-voltage curves are shown in Figure S17. Significant noise can be seen from both curves, suggesting the highly insulating nature of both MOFs. By performing linear fitting of the data as shown in the dashed line in the plot, slope of the current-voltage curve can be obtained, and the electrical conductivity of the material was estimated; see detailed procedures in our previous work.[13]

Fabrication of the Photothermoelectric Generator. The photothermoelectric generator (PTEG) was assembled using composite films that exhibited the highest measured *zT*. Among these, C/ZrBTBD10 and C/ZrBTBD-N5 were selected as the p-type and n-type components, respectively. The films were prepared using the previously described drop-casting method, with polyimide (PI) substrates serving as the support for each TE leg. Each film was cut into a rectangular shape measuring 7.5 mm in width and 30 mm in length. The p-type and n-type legs were alternately arranged on a flexible polyethylene terephthalate (PET) substrate and fixed in place using double-sided adhesive tape. Electrical connections between the legs were established using conductive silver paint, resulting in a series of five p-n junctions. Copper wires were connected to interface with external circuitry, and silver paste was applied at the contact points to reduce electrical resistance. Half part of the device was covered with aluminum foil to serve as a shaded region, while the remaining area was exposed to illumination. This setup enabled the formation of a thermal difference between the illuminated and shaded zones.

Measurement of thermoelectric, photothermal, and photothermoelectric properties. Thermoelectric properties were measured using a ZEM-3 instrument (ADVANCE RIKO Inc., Japan) under a helium atmosphere at 303 K. The in-plane Seebeck coefficient and electrical conductivity were recorded under temperature gradients of 20 K, 30 K, and 40 K. The Seebeck coefficient was determined by linear fitting of the voltage data. To minimize contact resistance

during electrical measurements, conductive silver paste was applied to both ends of the films. Thermal conductivity was measured at 25 °C using a Hot Disk TPS 2500S analyzer, which operates based on the transient plane source method. To meet the requirements of test module, thicker and larger-area composite films were fabricated using vacuum filtration. The testing protocols for these samples followed procedures reported in the literature.[2] During the photothermal characterization, the sample was irradiated with simulated sunlight generated by a 300 W xenon arc lamp (Yamashita Denso YSS-80S, AAA grade) to establish a temperature-increase profile. The system featured adjustable light intensity, allowing simulation of various illumination conditions. A thermal infrared camera (FLIR A50) was employed to continuously monitor and record the surface temperature evolution of the nanocomposite films during light exposure. The output performance of the photothermoelectric generator (PTEG) was evaluated under ambient conditions using a custom-built measurement platform. Current-voltage (I-V) and power-voltage (P-V) curves were recorded using a Keithley 2400 source meter, while a Keithley DAQ6510 precision voltmeter was used to measure the voltage generated under light irradiation. To further assess the practical applicability of the PTEG, it was affixed to a human forearm for in-situ testing.

Calculation of photothermal conversion efficiency.

The photothermal conversion Efficiency (η) was calculated based on the energy balance equation:

$$\eta = \frac{hS(T_{max} - T_{surr})}{I \cdot A \cdot (1 - 10^{-A_\lambda})}$$

In this framework, the equilibrium temperature (T_{max}) represents the steady-state temperature achieved under illumination, while the surrounding temperature (T_{surr}) serves as the baseline ambient temperature. The difference between these two values defines the effective temperature rise driving the thermal exchange. The energy input is characterized by the incident light intensity (I) and the geometric illumination area (A). A critical parameter in determining efficiency is the heat transfer coefficient (hS), which quantifies the system's ability to dissipate heat to the environment.

Table S1. Thermoelectric properties of CNT and CNT/MOF composite.

Sample	S [μV K^{-1}]	σ [S cm^{-1}]	PF [μW m^{-1} K^{-2}]
CNT	21.6 ± 0.5	2176.9 ± 86.8	111.2 ± 4.3
C/ZrBTB5	29.6 ± 2.2	2028.5 ± 49.0	186.5 ± 33.3
C/ZrBTB10	25.1 ± 2.5	1828.1 ± 59.9	122.3 ± 28.7
C/ZrBTB20	24.3 ± 2.8	1089.8 ± 90.3	75.2 ± 16.7
C/ZrBTB30	20.8 ± 0.9	788.0 ± 85.1	34.1 ± 4.3
C/ZrBTBD5	57.9 ± 1.5	1253.9 ± 54.8	409.5 ± 24.5
C/ZrBTBD10	62.9 ± 1.6	1172 ± 82.7	465.7 ± 39.5
C/ZrBTBD20	60.5 ± 1.8	667.5 ± 47.5	239.4 ± 21.7
C/ZrBTBD30	57.8 ± 2.0	508.0 ± 53.1	162.3 ± 17.9
C/ZrBTB-N2	52.1 ± 2.9	983.3 ± 47.9	269.9 ± 37.6
C/ZrBTB-N5	3.1 ± 5.8	770.4 ± 125.8	3.7 ± 2.7
C/ZrBTB-N8	–41.4 ± 2.0	1073.2 ± 82.3	183.3 ± 14.2
C/ZrBTB-N10	–36.3 ± 2.3	1568.2 ± 107.2	205.2 ± 10.7
C/ZrBTB-N14	–31.5 ± 2.8	1819.5 ± 46.8	175.3 ± 33.4
C/ZrBTBD-N2	–2.1 ± 12.9	965.5 ± 168.5	17.6 ± 13.7
C/ZrBTBD-N5	–54.2 ± 2.7	1242.4 ± 89.8	363.1 ± 16.7
C/ZrBTBD-N8	–43.0 ± 1.2	1340.8 ± 49.3	234.9 ± 15.8
C/ZrBTBD-N10	–37.8 ± 1.4	1609.0 ± 67.9	230.2 ± 16.1
C/ZrBTBD-N14	–31.1 ± 1.9	1745.0 ± 64.1	169.9 ± 27.1

Table S2. Thermoelectric properties of CNT/N719 dye composite.

Sample	S [μV K^{-1}]	σ [S cm^{-1}]	PF [μW m^{-1} K^{-2}]
CNT/N719 dye	–29.6 ± 13.5	1049.6 ± 150	100 ± 69.25

Table S3. Thermoelectric performance of CNT/MOF composite from previous reports and this work.

Samples	S [μV K^{-1}]	σ [S cm^{-1}]	PF [μW m^{-1} K^{-2}]	κ [W m^{-1} K^{-1}]	zT	Ref.
ZIF67@CNT	55.6	826	255.6	4.1	0.02	3
Ni-THT/SWCNT	40	613	98.1	0.8	0.037	4
Ni3 $(HITP)_2$ /CNT	40.7	150	24.9	0.7	0.008	5
Ni_3 $(HITP)_2$ /PEI-CNT	–30.5	90	8.4	0.6	0.0017	5
CNT/MIL-68 90%	38.6	97.1	14.2	0.2	0.025	6
CNT/MIL-68 50%	–49.3	153.2	46.4	0.2	0.071	6
SWCNTs/Fc@Cu-MOF	51.4	873.9	247.6	--	--	7
CNT/ZrBTB-BA (DCB)	58.7	1167.1	395.2	4.3	0.029	8
CNT/ZrBTB-BA (NMP)	–51.3	1254.9	330.8	3.8	0.027	8
C/ZrBTBD10 (This work)	62.9	1172	465.7	3.3	0.042	--
C/ZrBTBD-N5 (This work)	–54.2	1242.4	363.1	3.1	0.036	--

Table S4. Photothermal conversion efficiency (η) of C/ZrBTBD10 from previous reports and this work.

η (%)	Ref.
70.8%	[9]
46.8%	[10]
54.2%	[11]
30.6%	[12]
34.2%	This work

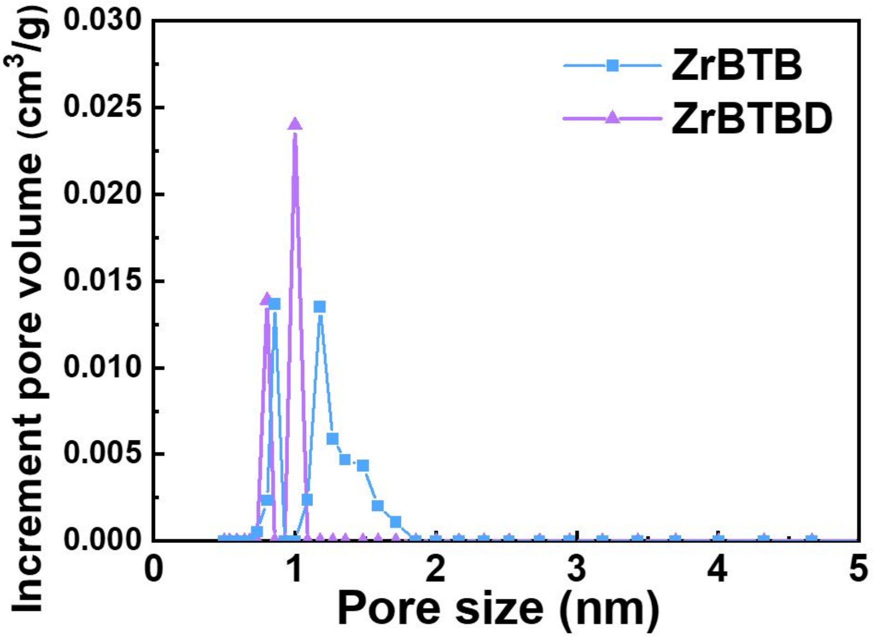

Figure S1. DFT pore size distributions of ZrBTB and ZrBTBD.

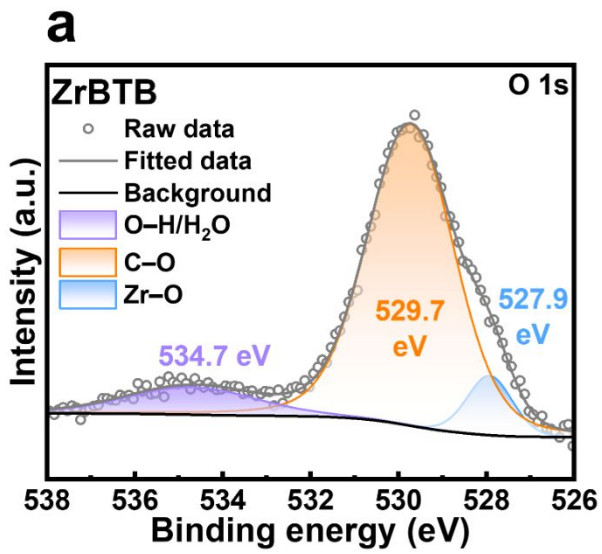

Figure S2. High-resolution XPS spectra of (a) ZrBTB and (b) ZrBTBD, collected in the region of O 1s.

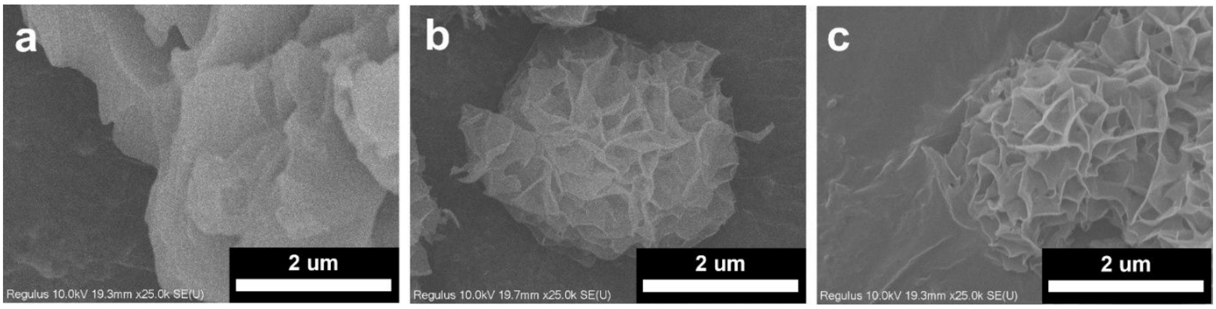

Figure S3. SEM images of (a) N719, (b) ZrBTB and (c) ZrBTBD.

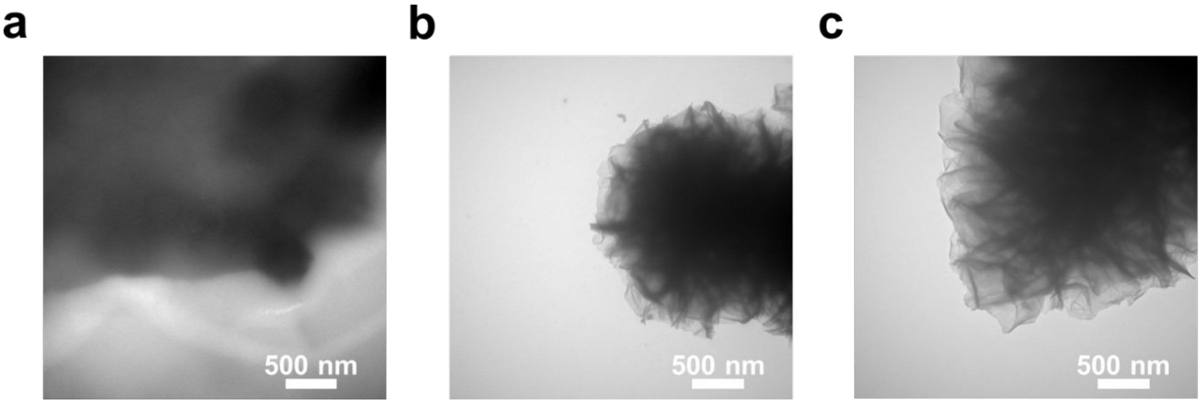

Figure S4. Low-magnification TEM images of (a) N719, (b) ZrBTB and (c) ZrBTBD.

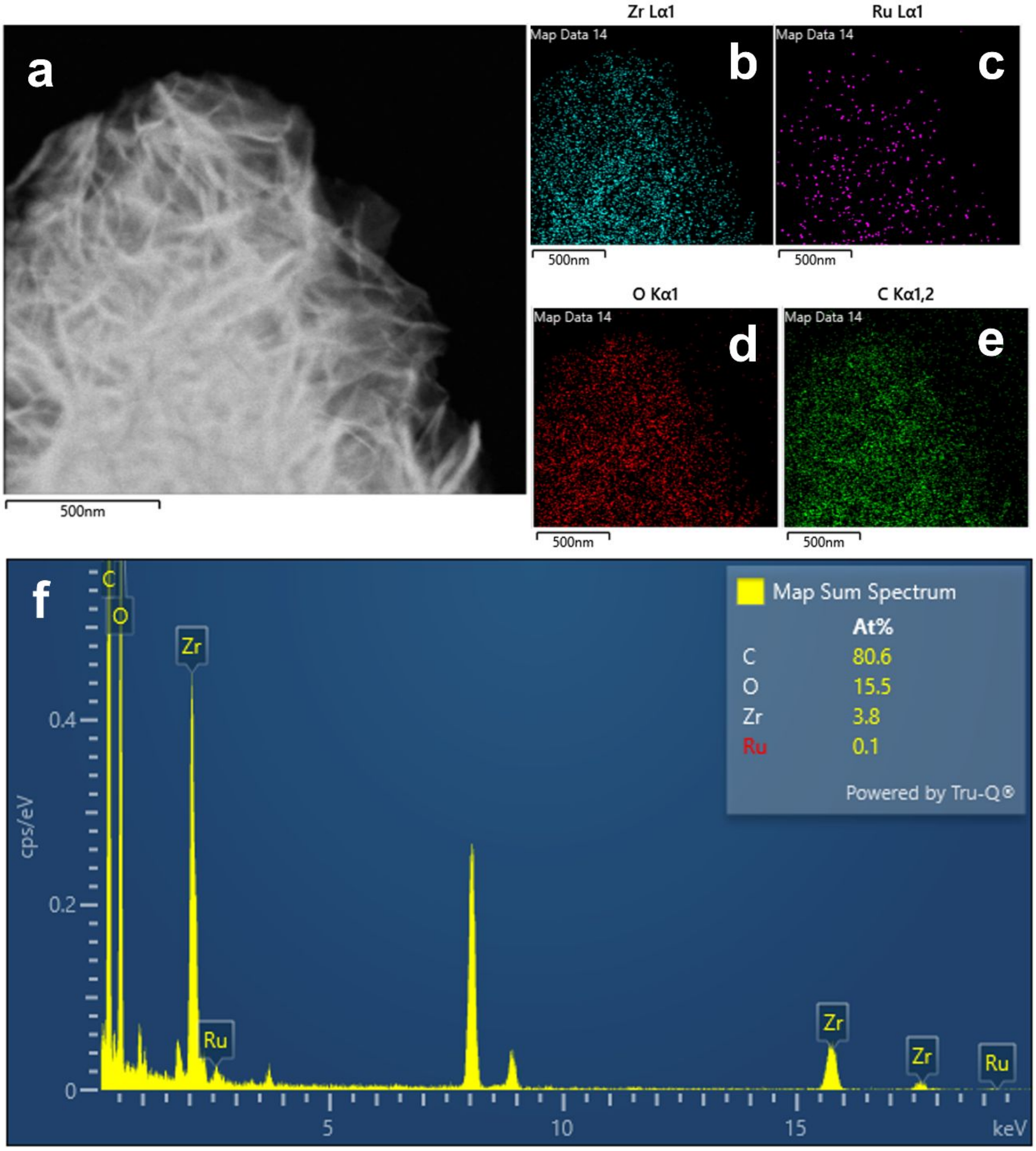

Figure S5. (a) TEM image of ZrBTBD, and corresponding EDS mapping data for (b) zirconium, (c) ruthenium, (d) oxygen and (e) carbon. (f) EDS spectrum of ZrBTBD.

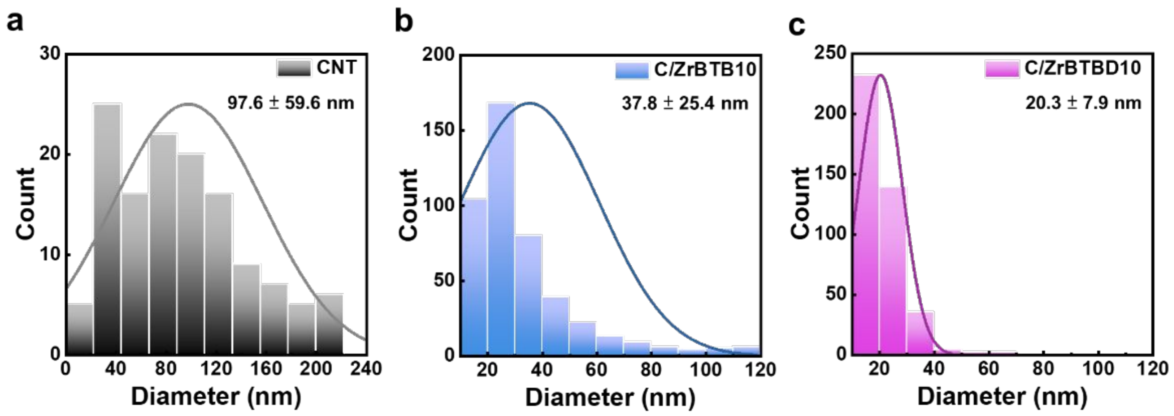

Figure S6. Corresponding CNT bundle size distributions of (a) CNT, (b) C/ZrBTB10, and (c) C/ZrBTBD10.

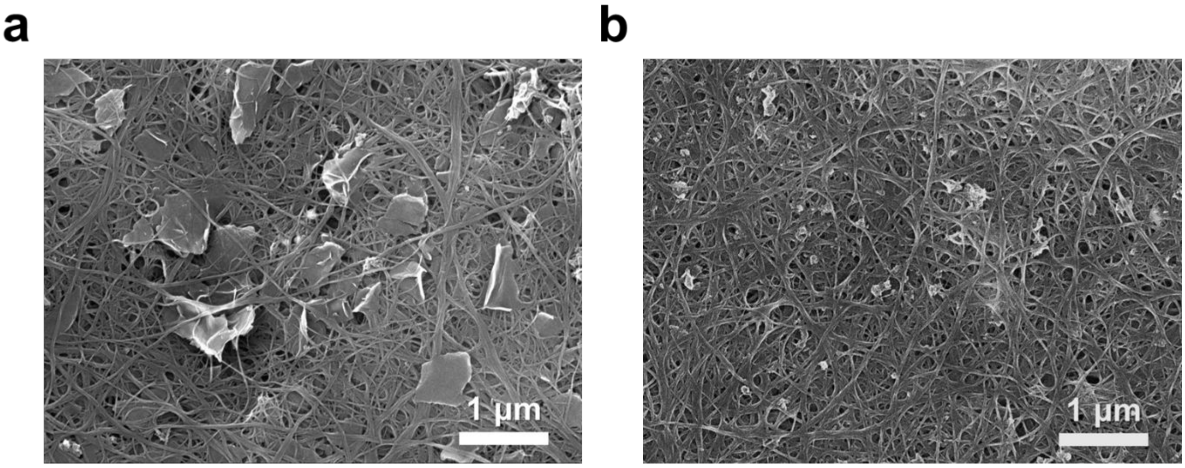

Figure S7. High-magnification SEM images of (a) C/ZrBTB-N5 (b) C/ZrBTBD-N5.

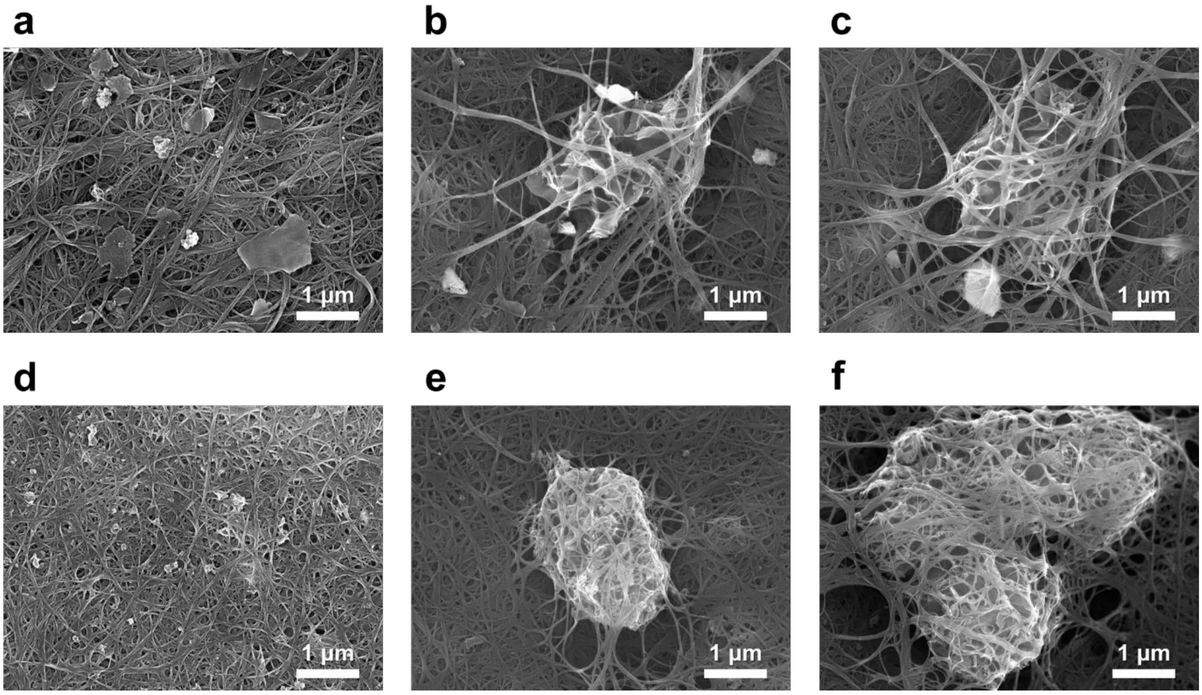

Figure S8. High-magnification SEM images of CNT/MOF composites (a) C/ZrBTB5, (b) C/ZrBTB20, (c) C/ZrBTB30, (d) CNT/ZrBTBD5, (e) CNT/ZrBTBD20 and (f) CNT/ZrBTBD30.

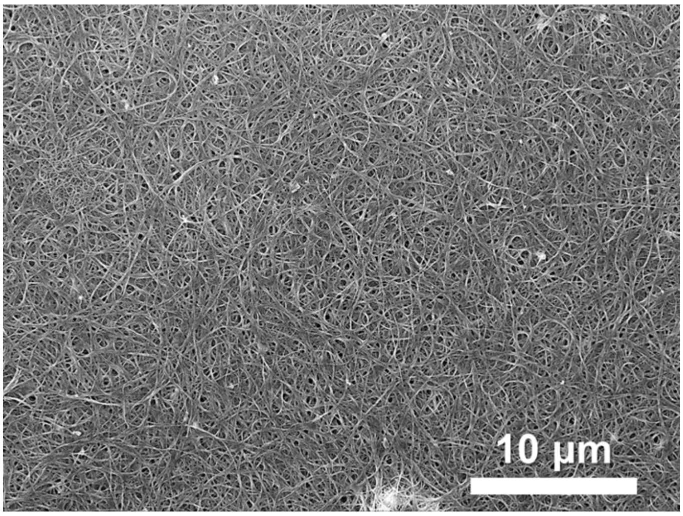

Figure S9. Low-magnification SEM images of pristine CNT.

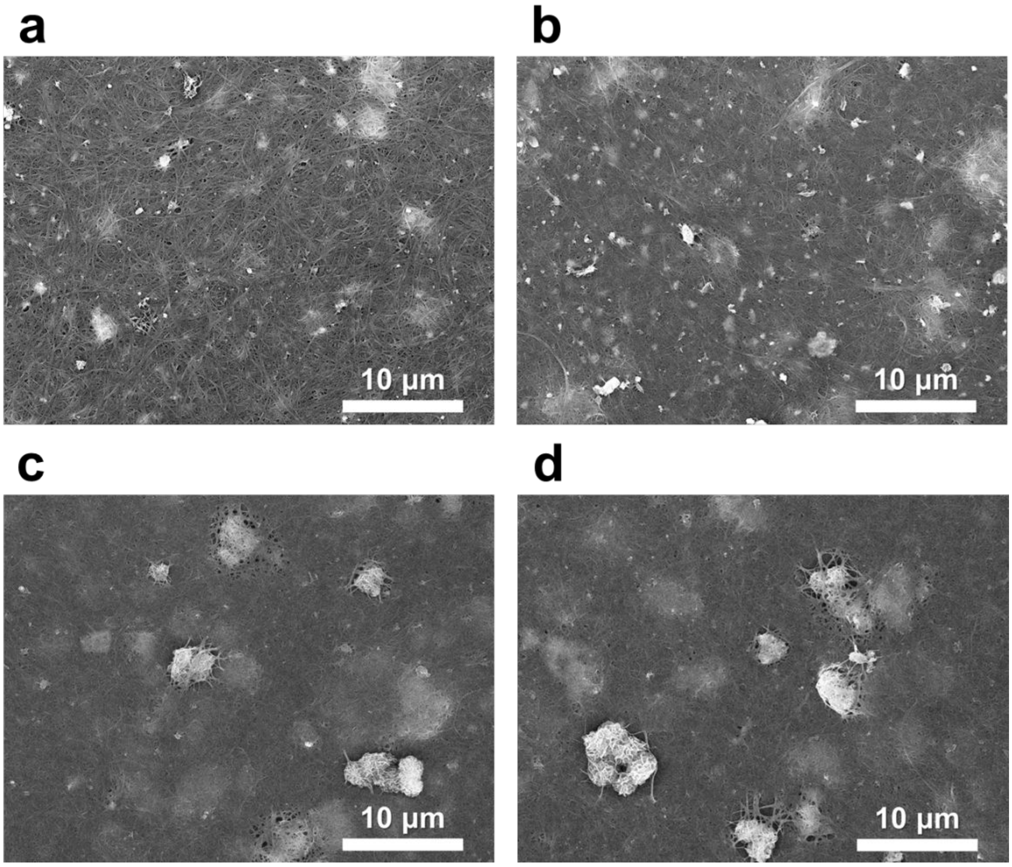

Figure S10. Low-magnification SEM images of CNT/MOF composites (a) C/ZrBTB5, (b) C/ZrBTB10, (c) C/ZrBTB20 and (d) C/ZrBTB30.

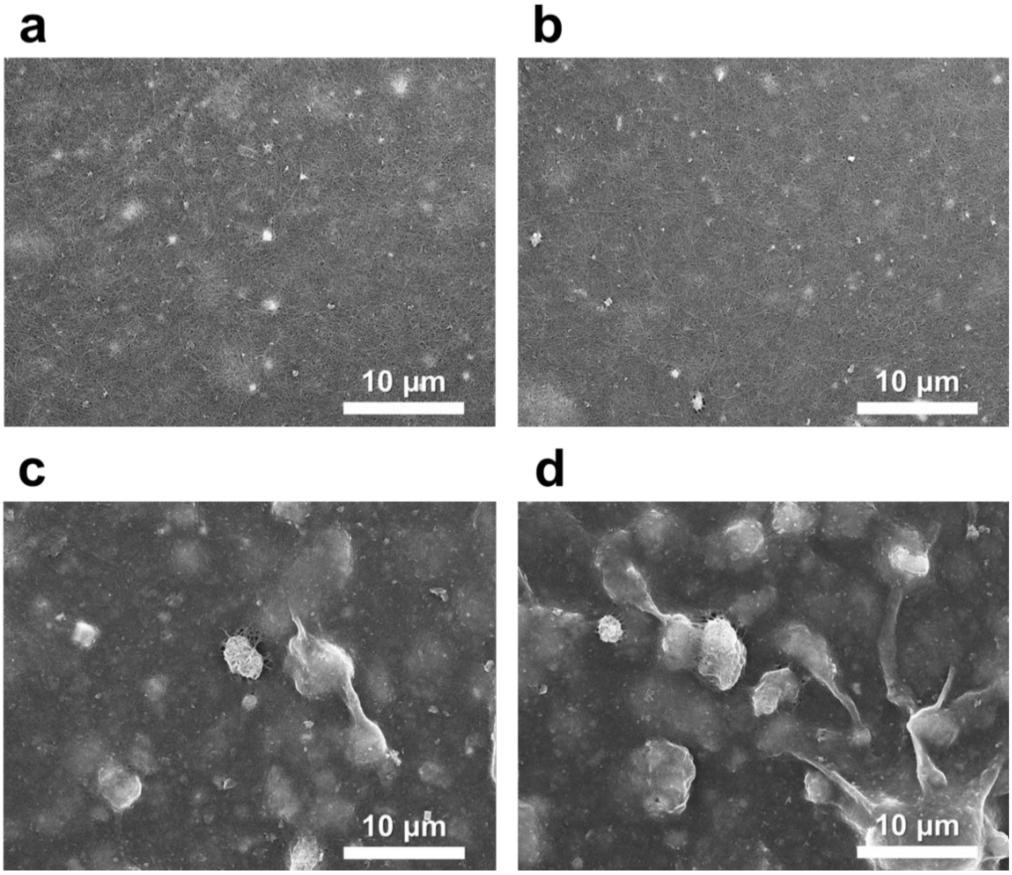

Figure S11. Low-magnification SEM images of CNT/MOF composites (a) C/ZrBTBD5, (b) C/ZrBTBD10, (c) C/ZrBTBD20 and (d) C/ZrBTBD30.

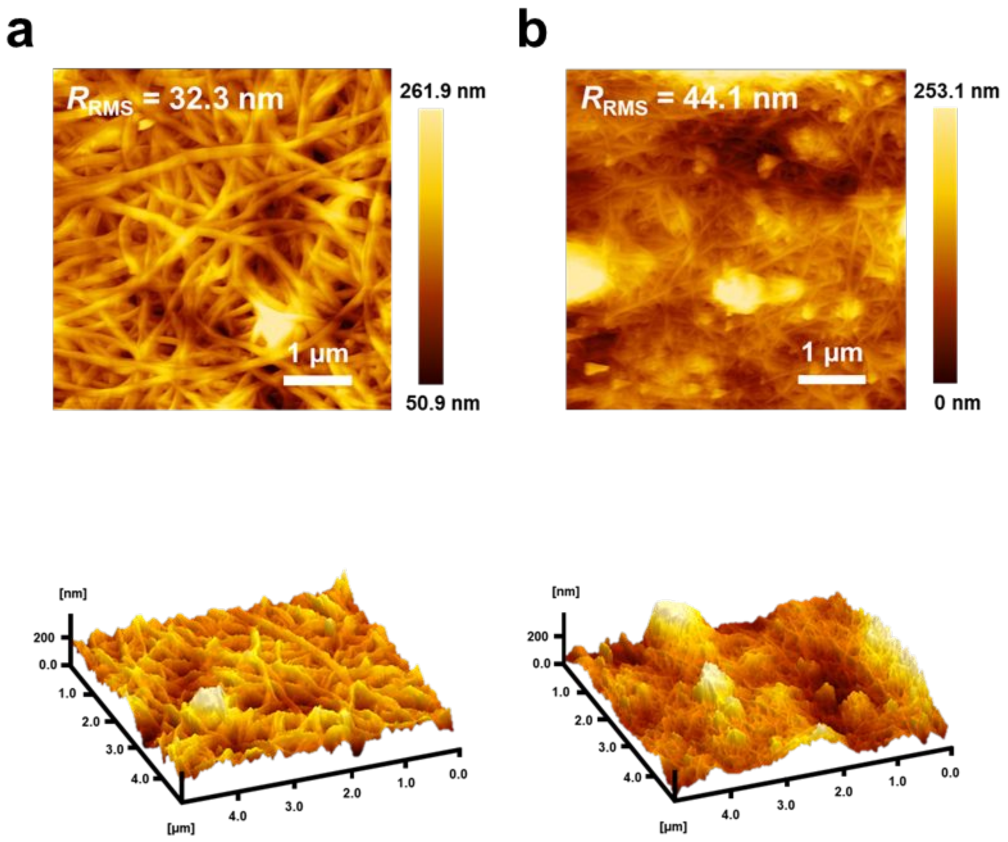

Figure S12. AFM images and corresponding 3D surface profiles of (a) pristine CNTs and (b) C/ZrBTBD10 composite films.

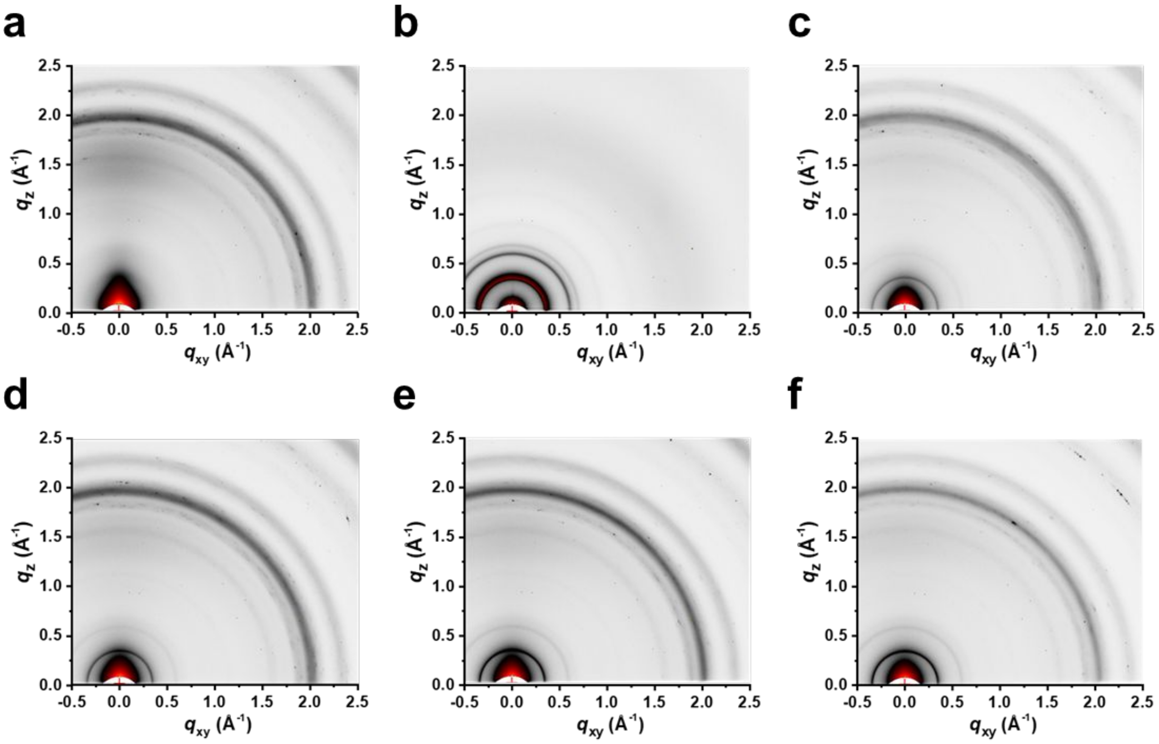

Figure S13. 2D GIXRD patterns of (a) pristine CNTs, (b) ZrBTB MOF, and (c–f) C/ZrBTB composites with increasing MOF loadings: (c) 5 wt %, (d) 10 wt %, (e) 20 wt %, and (f) 30 wt %.

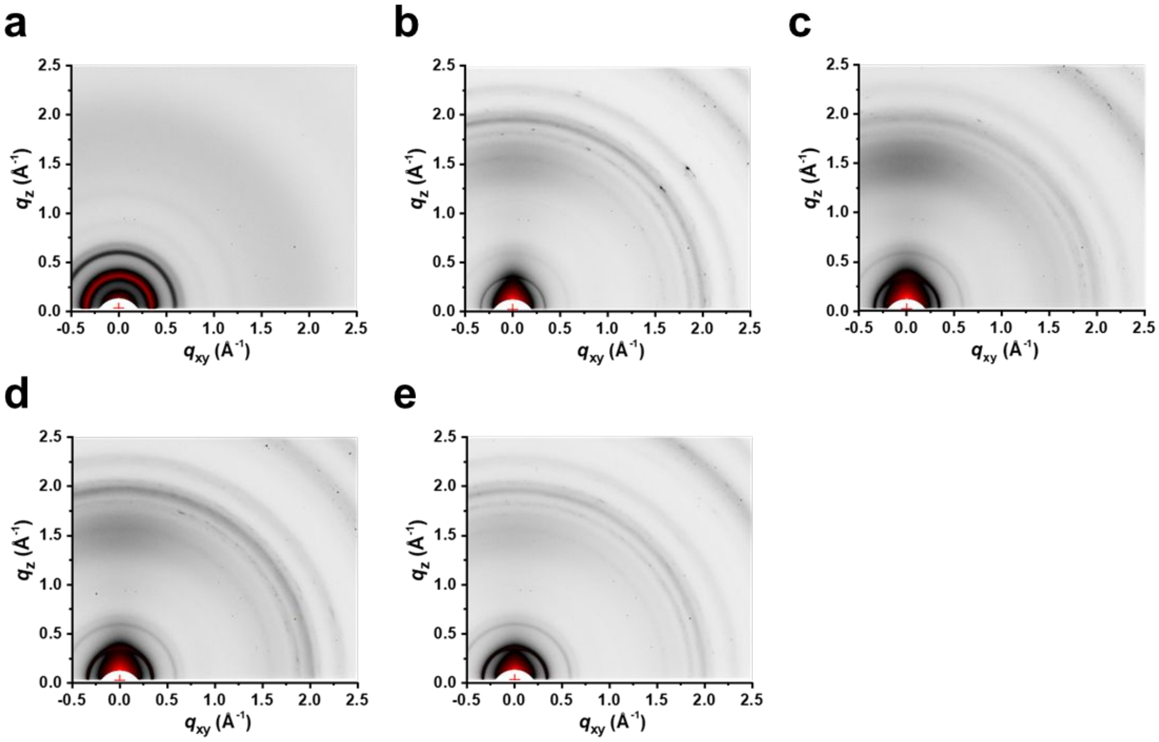

Figure S14. 2D GIXRD patterns of (a) ZrBTBD MOF, and (b–e) C/ZrBTBD composites with increasing MOF loadings: (b) 5 wt %, (c) 10 wt %, (d) 20 wt %, and (e) 30 wt %.

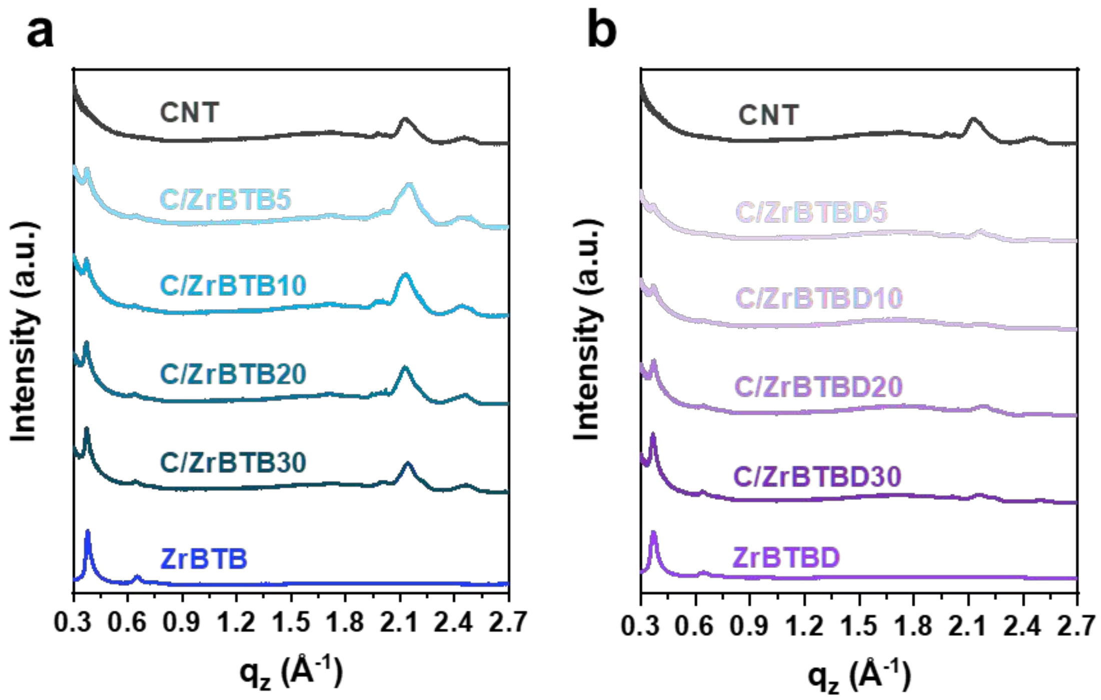

Figure S15. Out-of-plane integration of 2D GIXRD patterns of (a) C/ZrBTB composites, (b) C/ZrBTBD composites with varying MOF loadings.

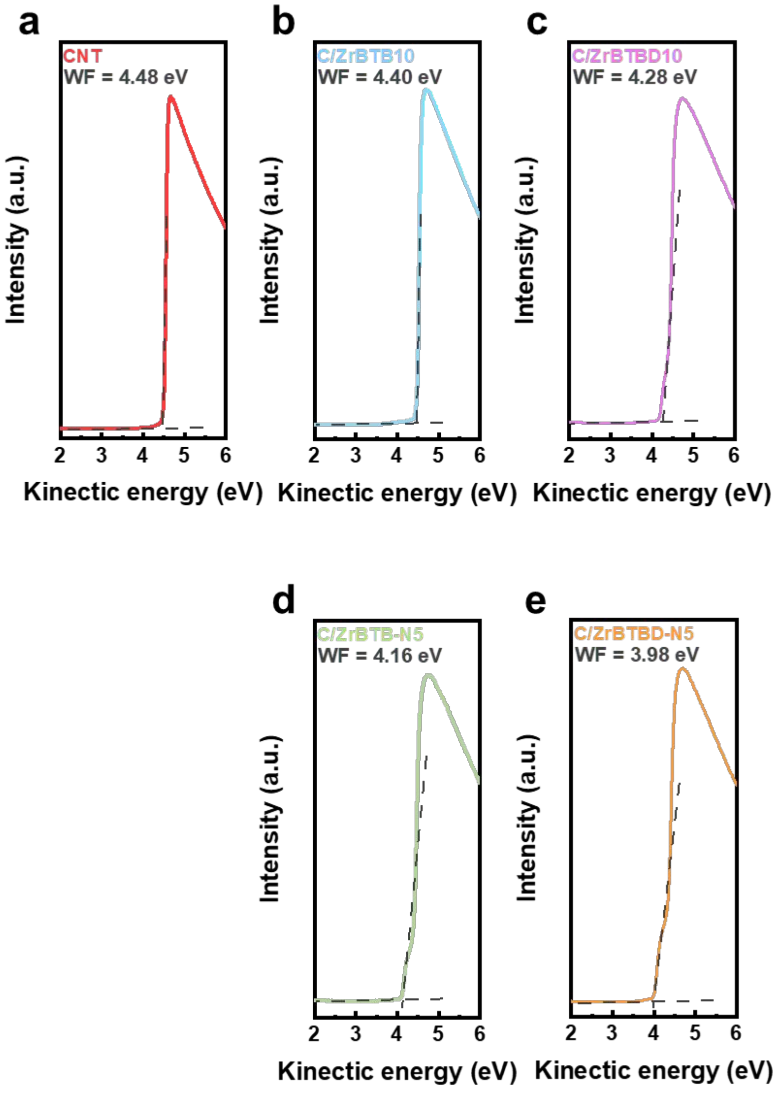

Figure S16. The UPS spectra and corresponding work function values of (a) CNT, (b) C/ZrBTB10, (c) C/ZrBTBD10, (d) C/ZrBTB-N5 and (e) C/ZrBTBD-N5 composites.

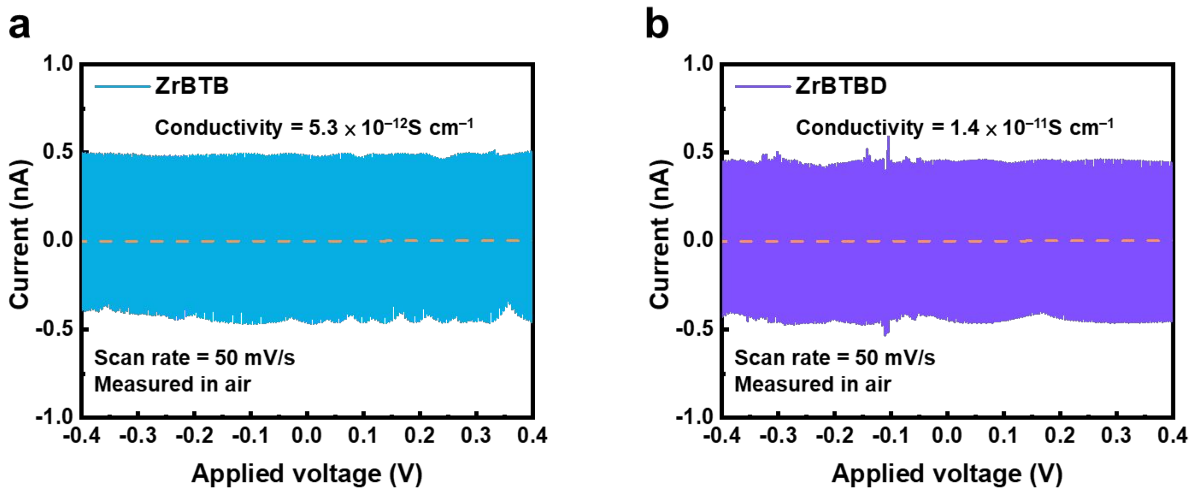

Figure S17. Current-voltage curves of pellets of (a) ZrBTB and (b) ZrBTBD

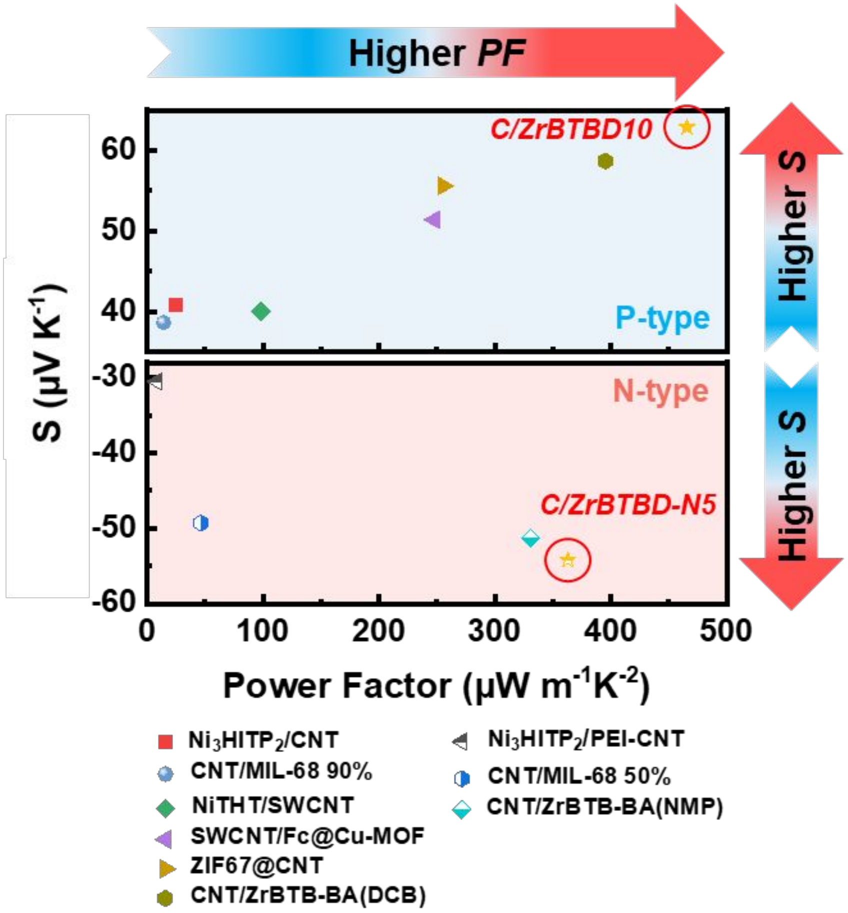

Figure S18. Comparative analysis of power factor values between this work and previous CNT/MOF-based thermoelectric materials, with p-type (top) and n-type (bottom) distinguished by Seebeck coefficient.

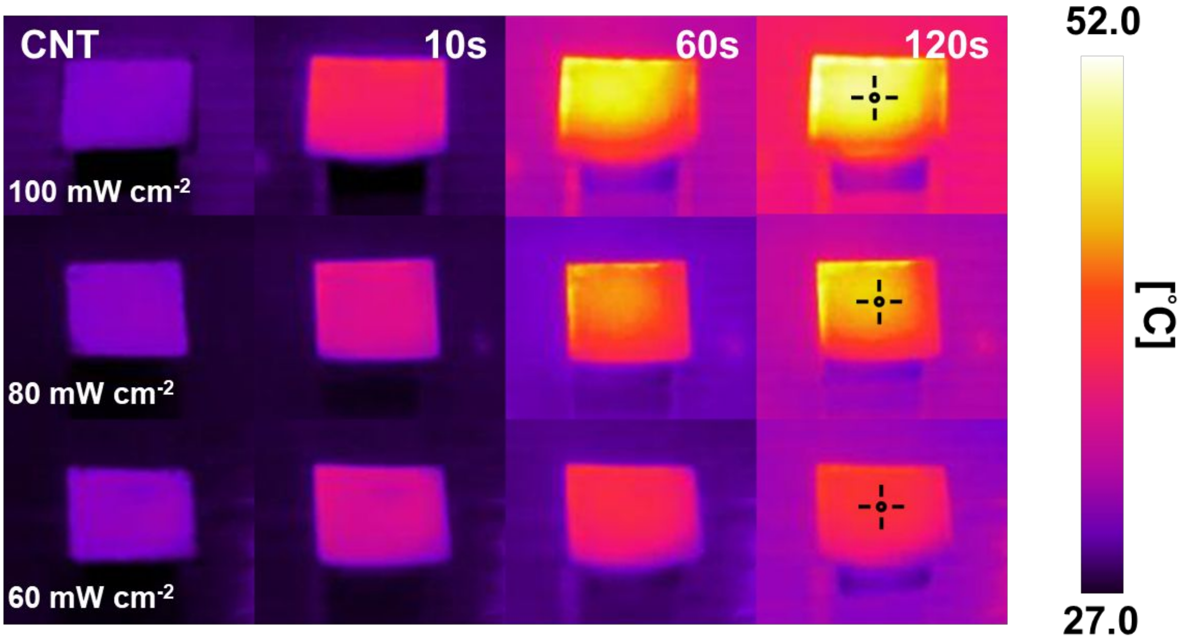

Figure S19. IR images of CNT under light irradiation at varying intensities: 100, 80, and 60 mW cm^{-2}.

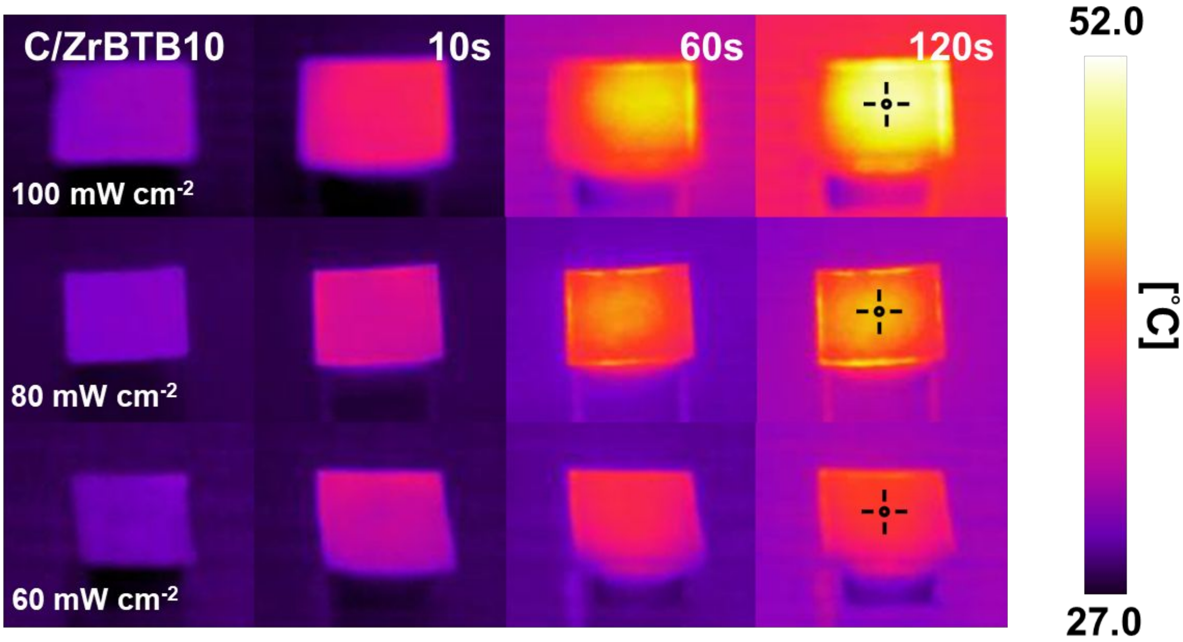

Figure S20. IR images of C/ZrBTB10 under light irradiation at varying intensities: 100, 80, and 60 mW cm^{-2}.

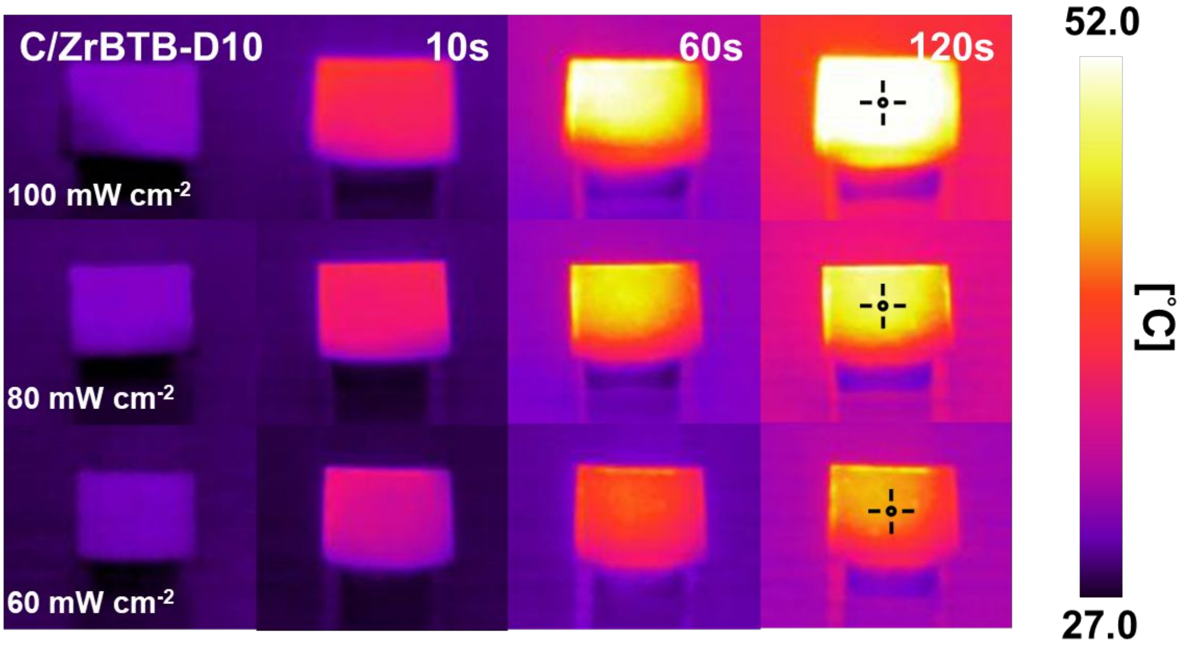

Figure S21. IR images of C/ZrBTBD10 under light irradiation at varying intensities: 100, 80, and 60 mW cm^{-2}.

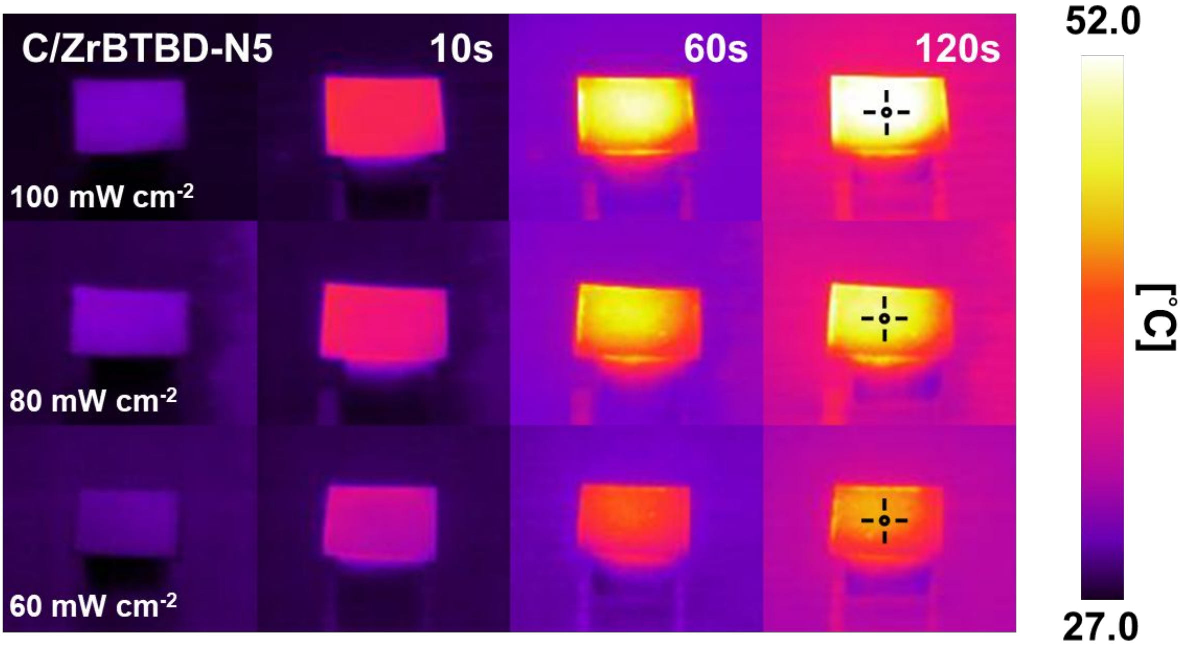

Figure S22. IR images of C/ZrBTBD-N5 under light irradiation at varying intensities: 100, 80, and 60 mW cm^{-2}.

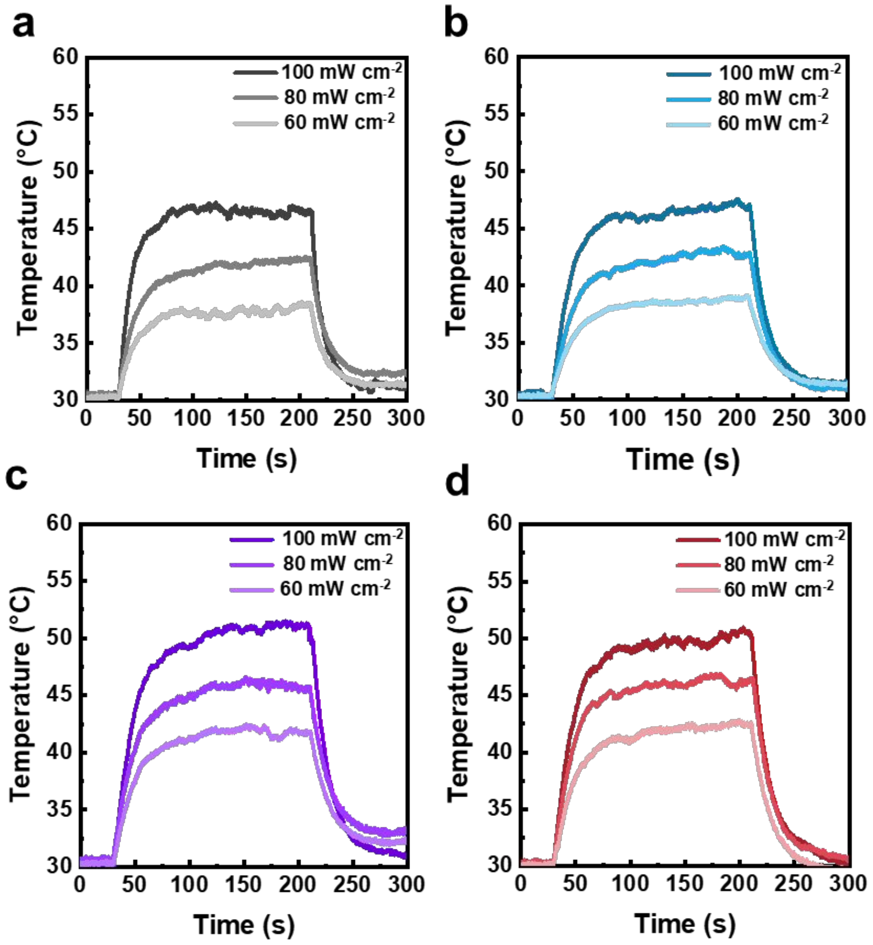

Figure S23. Time-dependent temperature profiles of (a) CNT, (b) C/ZrBTB10, (c) C/ZrBTBD10, and (d) C/ZrBTBD-N5 under light irradiation at different intensities (100, 80, and 60 mW cm^{-2}).

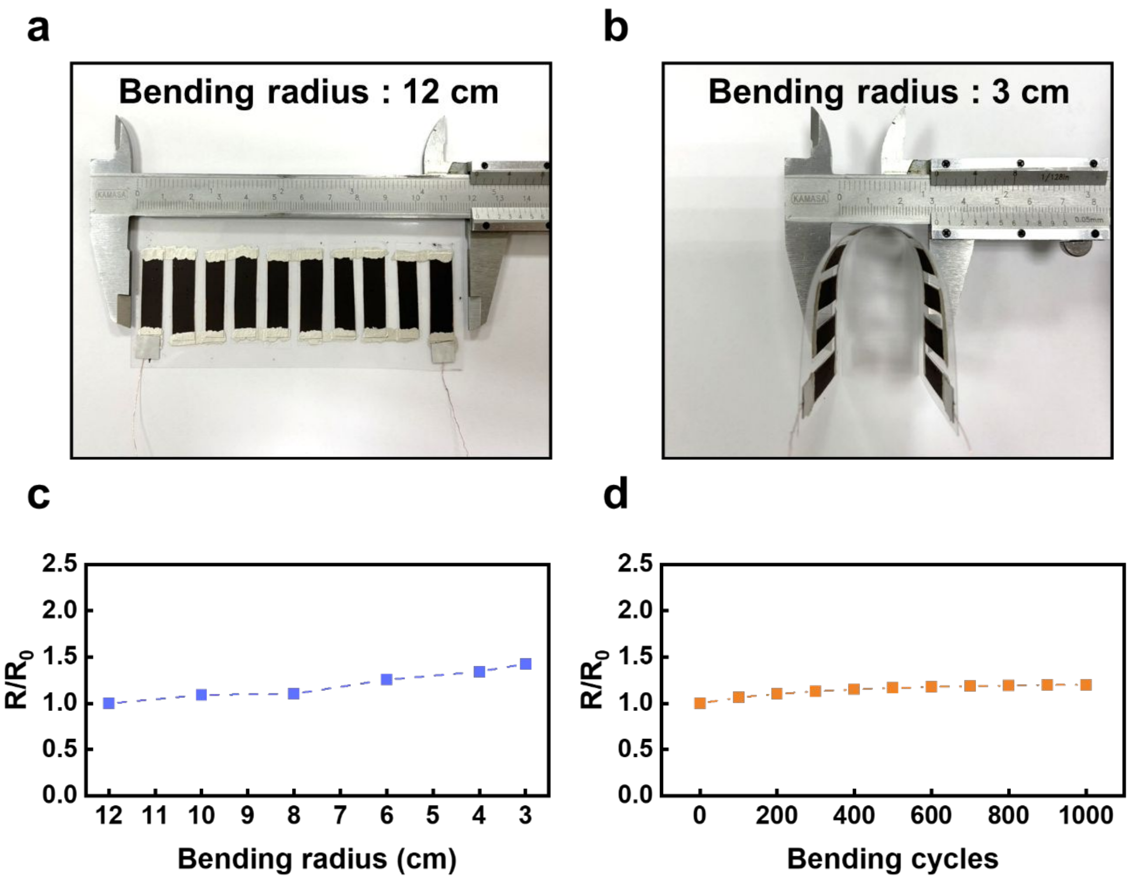

Figure S24. Photographic image of a PTEG device under a bending radius of (a) 12 cm, (b) 3 cm. The internal resistance analysis under different (c) bending radius and (d) bending cycles.

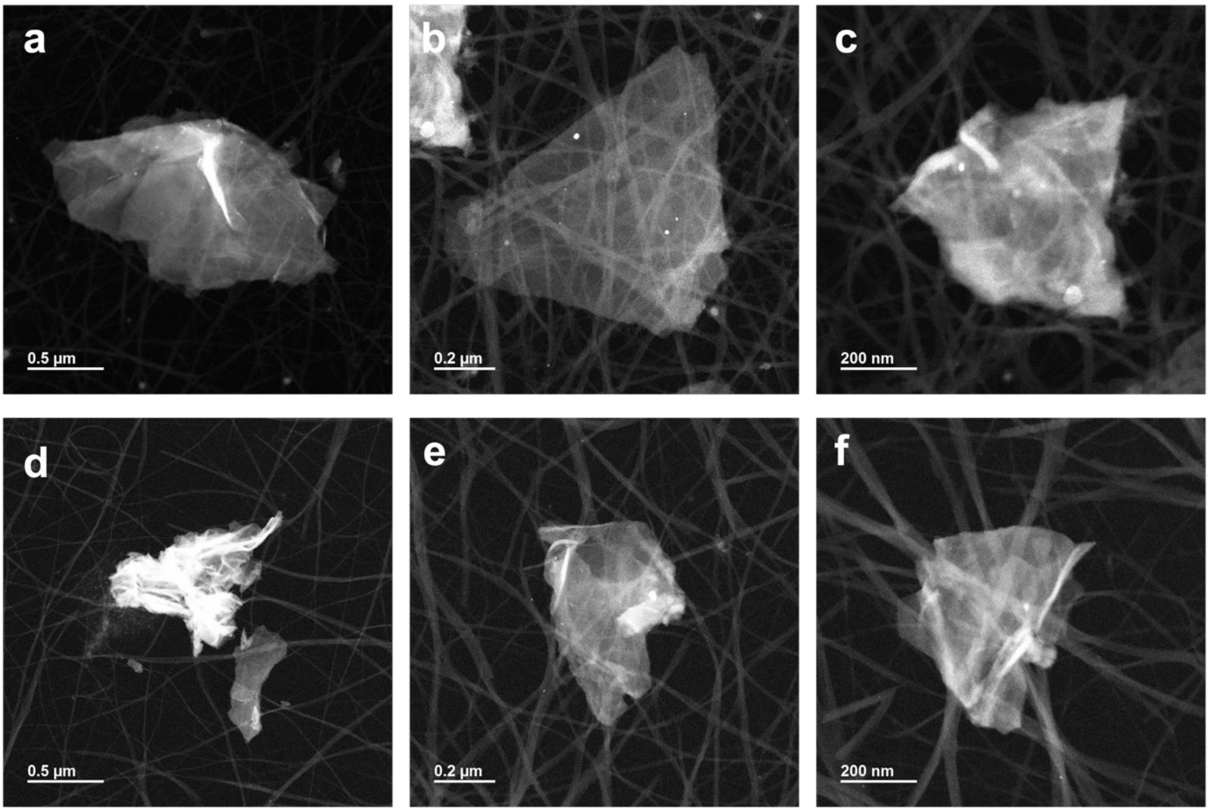

Figure S25. Scanning transmission electron microscope (STEM) dark-field images at different magnifications (a-c) C/ZrBTB, (d-f) C/ZrBTBD.

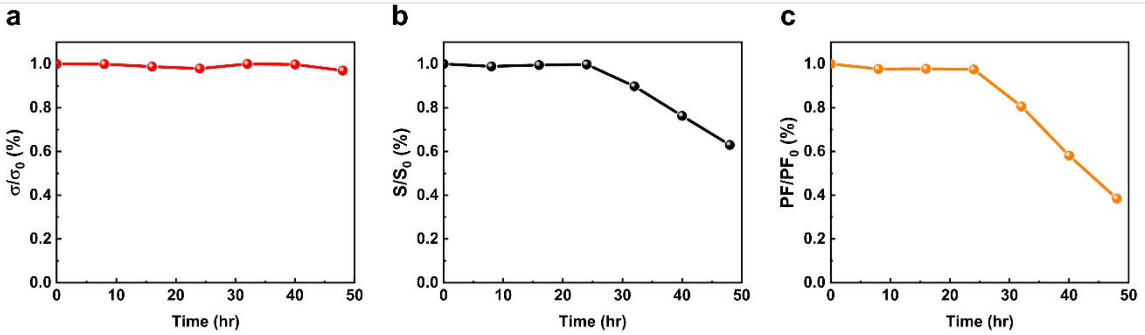

Figure S26. Stability of C/ZrBTBD-N5 under ambient conditions: (a) electrical conductivity, (b) Seebeck coefficient, and (c) power factor.

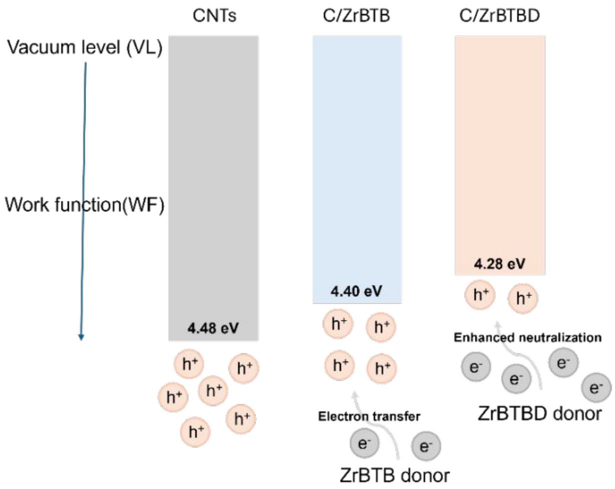

Figure S27. Schematic illustration of energy level alignment and charge transfer mechanism in CNTs, C/ZrBTB, and C/ZrBTBD composites.

REFERENCES

(1) Chen, Y.-L.; Shen, C.-H.; Huang, C.-W.; Kung, C.-W. Terbium-modified two-dimensional zirconium-based metal–organic frameworks for photoluminescence detection of nitrite. *Mol. Syst. Des. Eng.* **2023**, *8* (3), 330-340, 10.1039/D2ME00214K. DOI: 10.1039/D2ME00214K.
(2) Lin, M.-H.; Mohamed, M. G.; Lin, C.-J.; Sheng, Y.-J.; Kuo, S.-W.; Liu, C.-L. Achieving High zT with Carbon Nanotube/Conjugated Microporous Polymer Thermoelectric Nanohybrids by Meticulous Molecular Geometry Design. *Adv. Funct. Mater.* **2024**, *34* (45), 2406165. DOI: https://doi.org/10.1002/adfm.202406165 (acccessed 2024/12/08).
(3) Xue, Y.; Zhang, Z.; Zhang, Y.; Wang, X.; Li, L.; Wang, H.; Chen, G. Boosting thermoelectric performance by in situ growth of metal organic framework on carbon nanotube and subsequent annealing. *Carbon* **2020**, *157*, 324-329. DOI: https://doi.org/10.1016/j.carbon.2019.10.049.
(4) Chen, Z.; Cui, Y.; Liang, L.; Wang, H.; Xu, W.; Zhang, Q.; Chen, G. Flexible film and thermoelectric device of single-walled carbon nanotube@conductive metal-organic framework composite. *Mater. Today Nano* **2022**, *20*, 100276. DOI: https://doi.org/10.1016/j.mtnano.2022.100276.
(5) Qi, X.; Wang, Y.; Li, K.; Wang, J.; Zhang, H.-L.; Yu, C.; Wang, H. Enhanced electrical properties and restrained thermal transport in p- and n-type thermoelectric metal–organic framework hybrids. *J. Mater. Chem. A* **2021**, *9* (1), 310-319, 10.1039/D0TA10051J. DOI: 10.1039/D0TA10051J.
(6) Lin, M.-H.; Hsu, C.-H.; Kang, D.-Y.; Liu, C.-L. Correlating framework structures and thermoelectric performance of metal–organic framework/carbon nanotube thermoelectric hybrids with n–p type inversion. *Chem. Eng. J.* **2024**, *485*, 149732.
(7) Tian, G.; Zhang, J.; Li, Z.; Guo, L.; Fu, P.; Tang, C.; Tsui, C.; Zhang, Y.; Du, F. Structural regulation of ferrocene-doping Cu-MOFs for enhancing thermoelectric properties of single-walled carbon nanotube-based composite films. *J. Power Sources* **2025**, *630*, 236121. DOI: https://doi.org/10.1016/j.jpowsour.2024.236121.
(8) Lin, C.-Y.; Chang, J.-W.; Lin, M.-H.; Wu, K.-C.; Hong, S.-H.; Lin, J.-M.; Kung, C.-W.; Liu, C.-L. Carbon nanotube/two-dimensional metal–organic framework composites with enhanced thermoelectric performances for thermoelectric generators. *Chem. Eng. J.* **2025**, *521*, 166861. DOI: https://doi.org/10.1016/j.cej.2025.166861.
(9) Zhou, L.; Wang, X.; Zhang, J.; Yang, S.; Hao, K.; Gao, Y.; Li, D.; Li, Z. Self-suspended carbon nanotube/polyimide composite film with improved photothermal properties. *J. Appl. Phys.* **2020**, *127* (20).
(10) Alrahili, M. Light to heat conversion efficiency of single-walled carbon nanotubes. *J. Taibah Univ. Sci.* **2022**, *16* (1), 923-932.
(11) Wei, C.; Jin, X.; Wu, C.; Brozovic, A.; Zhang, W. Carbon spheres with high photothermal conversion efficiency for photothermal therapy of tumor. *Diamond Relat. Mater.* **2022**, *126*, 109048.
(12) Yang, D.; Zhou, B.; Han, G.; Feng, Y.; Ma, J.; Han, J.; Liu, C.; Shen, C. Flexible transparent polypyrrole-decorated MXene-based film with excellent photothermal energy conversion performance. *ACS Appl. Mater. Interfaces* **2021**, *13* (7), 8909-8918.
(13) Tsai, M.-D.; Chen, Y.-L.; Chang, J.-W.; Yang, S.-C.; Kung, C.-W. Sulfonate-Functionalized Two-Dimensional Metal–Organic Framework as a “Dispersant” for Polyaniline to Boost Its Electrochemical Capacitive Performance. *ACS Appl. Energy Mater.* **2023**, *6* (21), 11268-11277. DOI: 10.1021/acsaem.3c02155.
